# Nach Is a Novel Subgroup at an Early Evolutionary Stage of the CNC-bZIP Subfamily Transcription Factors from the Marine Bacteria to Humans

**DOI:** 10.3390/ijms19102927

**Published:** 2018-09-26

**Authors:** Yu-Ping Zhu, Meng Wang, Yuancai Xiang, Lu Qiu, Shaofan Hu, Zhengwen Zhang, Peter Mattjus, Xiaomei Zhu, Yiguo Zhang

**Affiliations:** 1 The Laboratory of Cell Biochemistry and Topogenetic Regulation, College of Bioengineering and Faculty of Sciences, Chongqing University, No. 174 Shazheng Street, Shapingba District, Chongqing 400044, China; zhuyupingzhuyu@163.com (Y.-P.Z.); 20151901005@cqu.edu.cn (M.W.); yuancaix@126.com (Y.X.); qiulu99999@163.com (L.Q.); hufan2441@163.com (S.H.); 2 Institute of Neuroscience and Psychology, School of Life Sciences, University of Glasgow, 42 Western Common Road, Glasgow G22 5PQ, Scotland, UK; zzhengwen@hotmail.co.uk; 3 Department of Biochemistry, Faculty of Science and Engineering, Åbo Akademi University, Artillerigatan 6A, III, BioCity, FI-20520 Turku, Finland; pmattjus@abo.fi; 4 Shanghai Center for Quantitative Life Science and Department of Physics, Shanghai University, 99 Shangda Road, Shanghai 200444, China; xiaomeizhu@yahoo.com

**Keywords:** Nach, CNC, bZIP transcription factor, interaction network, evolution, transmembrane, topobiology, moving membrane-proteins, degron, suicidon, redox stress, Nrf1, Nrf2, ATF6, Jun

## Abstract

Normal growth and development, as well as adaptive responses to various intracellular and environmental stresses, are tightly controlled by transcriptional networks. The evolutionarily conserved genomic sequences across species highlights the architecture of such certain regulatory elements. Among them, one of the most conserved transcription factors is the basic-region leucine zipper (bZIP) family. Herein, we have performed phylogenetic analysis of these bZIP proteins and found, to our surprise, that there exist a few homologous proteins of the family members Jun, Fos, ATF2, BATF, C/EBP and CNC (cap’n’collar) in either viruses or bacteria, albeit expansion and diversification of this bZIP superfamily have occurred in vertebrates from metazoan. Interestingly, a specific group of bZIP proteins is identified, designated Nach (*N*rf *a*nd *C*NC *h*omology), because of their strong conservation with all the known CNC and NF-E2 p45 subunit-related factors Nrf1 and Nrf2. Further experimental evidence has also been provided, revealing that Nach1 and Nach2 from the marine bacteria exert distinctive functions, when compared with human Nrf1 and Nrf2, in the transcriptional regulation of antioxidant response element (ARE)-battery genes. Collectively, further insights into these Nach/CNC-bZIP subfamily transcription factors provide a novel better understanding of distinct biological functions of these factors expressed in distinct species from the marine bacteria to humans.

## 1. Introduction

The evolutionarily conserved sequences across species may be attributed to at least two main reasons, i.e., conserved helices (such as in 16S rRNA [1]) and consensus regulatory elements existing especially among developmental process-related transcription factor (TF) genes in living organisms [2]. To ensure that only the fittest of life forms can survive and also maintain a robust homoeostasis being established during the nature selection [3], their transcriptional networks that are composed of distinct transcription factor families play essential roles in regulating the expression of different sets of cognate target genes [4]. Such ability of TFs is manifested by their specific *cis*-regulatory DNA sequences, e.g., antioxidant response elements (AREs) and activating protein-1 (AP-1)-binding site, in order to control the transcriptional expression of cognate target genes and also display relevant functional performances in many ways [5,6]. Therefore, here analyzing the evolutionarily conserved blocks (i.e., domains or consensus motifs) within TFs is an effective way to elucidate the architecture of regulatory networks and their relationships in different species [2].

Notably, one of the most conserved TFs is the basic-region leucine zipper (bZIP) superfamily. They are involved in the transcriptional regulation of differential subsets of target genes by forming homo- and hetero-dimers with their cognate partners before binding their specific *cis*-regulatory elements (e.g., ARE or AP-1) in the promoter regions of these genes. The transcriptional networks formed by distinct arrays of such dimerization of the bZIP superfamily are known to play vital roles in cell division, proliferation, differentiation, maintenance, and other life processes, particularly in multicellular organisms [7]. Conversely, both structural and functional deficiencies in some bZIP factors can result in various diseases, including cancer, autoimmune, and inflammatory diseases, and defaults in many other pathological processes [8,9,10,11]. Furthermore, the highly conserved bZIP protein family is predominantly determined by the founding domain (i.e., BRLZ), that is composed of the basic-region (BR) and leucine zipper (LZ) repeats, with 60–80 amino acids (aa) in length [12]. The basic-region comprises an approximately 16-aa consensus sequence, which is responsible for a putative nuclear localization signal (NLS) and DNA-binding activity to gain access to target genes. Besides, the LZ region is composed of heptad repeats of leucine or other bulky hydrophobic residues exactly occupied at the “*d*” positions, and mediates dimerization of bZIP proteins [13,14].

Currently, with the availability of whole genome sequences from distinct species, an ever- increasing number of bZIP proteins are identified as key players in defending against abiotic stresses in plants, including Arabidopsis [15], rice [16], apple [17], and maize [18]. In animals, a similar bZIP superfamily also appears to have originated throughout the eukaryotic evolution process before the dawn of the Metazoa. This is supported by the fact that some of the highly conserved bZIP family proteins have emerged in the protozoa, such as choanoflagellate (*Monosiga brevicollis*) and protist (*Capsaspora owczarzaki*) [19], in addition to the presence of orthologues in the Metazoa. Immediately, with accumulating analyses of the elaborate bZIP-mediated transcriptional networks within distinct eukaryotes, their evolutionary process had also been investigated in animals [19,20], fungi [21], and plants [22]. For example, the bZIP family in Metazoa was initially thought to be evolved from a last single putative common progenitor eukaryotic gene, which had undergone multiple independent expansions and three major evolutionary periods [23]. Consequently, three identifiable ancestral opisthokont bZIP proteins ATF6, ATF2-sko1, and Jun-CGN4 were found [23]. Nonetheless, the evolutionary origin of the bZIP superfamily remains elusive, in particular, the evolution of the CNC (cap’n’collar)-bZIP subfamily is limited.

The CNC-bZIP subfamily contains a founding consensus CNC domain situated in front of its BRLZ domain, which is uniquely distinctive from other bZIP subfamilies. Among its previously assigned members are nuclear factor-erythroid 2 (NF-E2) p45 and related factors (Nrfs), along with two transcriptional repressors Bach1 and Bach2 in vertebrates, and the *Caenorhabditis elegans* protein Skn-1 [24], in addition to the founding *Drosophila melanogaster* Cnc protein [25]. Here, we discovered an early-evolved subgroup of CNC-bZIP proteins, designated “Nach”, because of their strong conservation with all those known Nrf/CNC-bZIP proteins. Interestingly, Nach1 and Nach2 from marine bacteria play a distinctive role, when compared to human Nrf1 and Nrf2, in transcriptionally regulating expression of ARE-driven genes. Our phylogenetic analysis has demonstrated that the Nach/CNC-bZIP subfamily shares an early evolutionary stem with their partner Maf subfamily, implying that they originated from a common ancestor. In this study, the membrane-bound bZIP proteins were also identified and the interaction networks of bZIPs in the human were further analyzed. Moreover, the phylogenetic tree of all 53 bZIP proteins in humans was also constructed. Notably, distinct or opposing changes in some nodes within the two interaction networks, which are composed of all the human bZIP proteins and also converged on a hub of Nrf1α, were determined following knockout of Nrf1α or induction of its protein expression by tetracycline treatment of HEK293C^Nrf1α^ cells. Taken altogether, the regulatory network demonstrates the importance of Nrf1α hub with the rest of bZIP genes.

## 2. Results

### 2.1. Species Distribution and Phylogenetic Analysis of bZIP Transcription Factors

**Figure 1:**
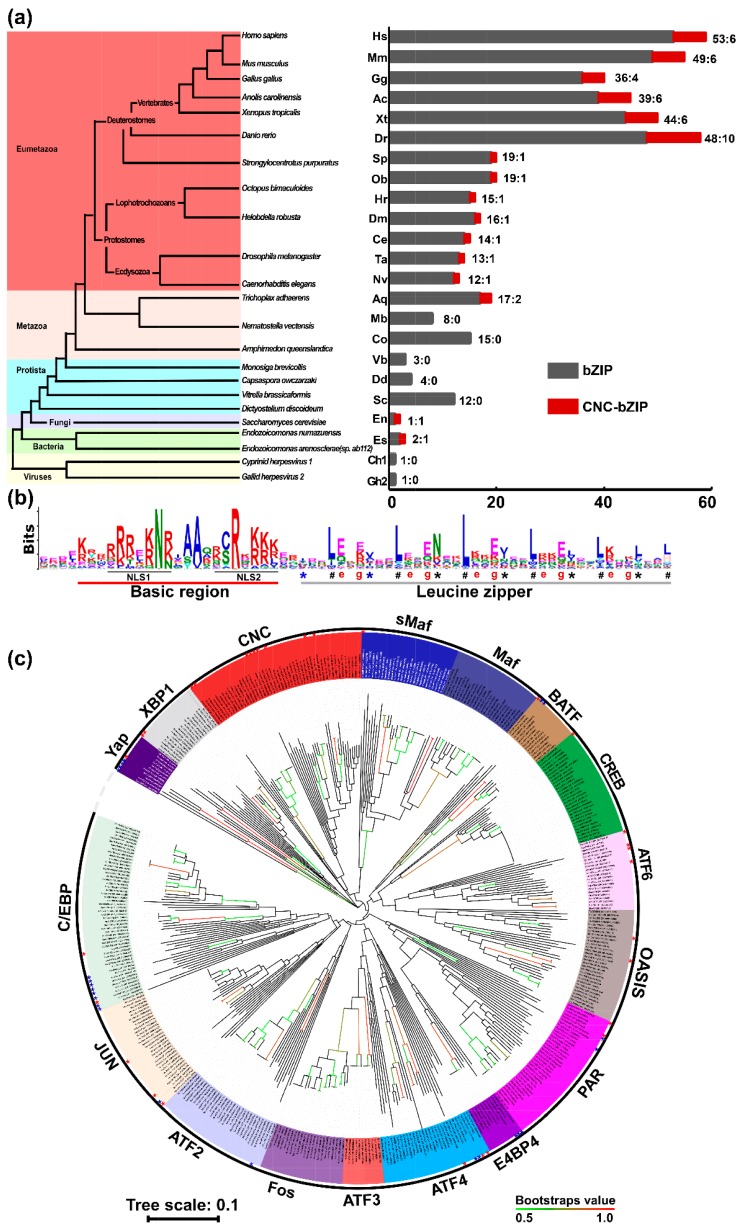
Species distribution and phylogenetic analysis of the bZIP transcription factors. (**a**) The left panel shows distinct evolutionary status of representative species with distinct organisms, while the right graph illustrates discrepant distribution of total bZIP (in black column) and particular Nach/CNC-bZIP proteins (in the dark red column) existing in each of different species. They include *Gallid herpesvirus 2* (Gh2), *Cyprinid herpesvirus 1* (Ch1), *Endozoicomonas numazuensis* (En), *Endozoicomonas arenosclerae* (or sp. ab112) (Ea/Es), *Dictyostelium discoideum* (Dd), *Vitrella brassicaformis* (Vb), *Saccharomyces cerevisiae* (Sc), *Monosiga brevicollis* (Mb), *Capsaspora owczarzaki* (Co), *Trichoplax adhaerens* (Ta), *Amphimedon queenslandica* (Aq), *Nematostella vectensis* (Nv), *Caenorhabditis elegans* (Ce), *Drosophila melanogaster* (Dm), *Helobdella robusta* (Hr), *Octopus bimaculoides* (Ob), *Strongylocentrotus purpuratus* (Sp), *Danio rerio* (Dr), *Xenopus tropicalis* (Xt), *Gallus gallus* (Gg), *Anolis carolinensis* (Ac), *Mus musculus* (Mm), and *Homo sapiens* (Hs). (**b**) Shows a color Logo image obtained from the MEME analysis of both the basic-region (BR) and leucine zipper (LZ) domains within 441 of bZIP transcription factors, in which the location of a bipartite nuclear localization signal (NLS, that is composed of two parts NLS1 and NLS2) is underlined, whilst the “*a*” and “*d*” positions at the putative helixes folded by six heptad repeats are indicated by different symbols * and #, respectively; The red letter e and g represent the fifth and seventh positions in heptad repeats of LZ region, respectively. (**c**) The neighbor-joining (NJ) phylogenetic tree of BRLZ domains within 441 bZIP proteins across 23 representative species was drawn by MEGA 6.0 with 1000 bootstrap. The more than 50% of bootstrap values were shown by from green to red clades; the green clades stand for minimum bootstrap values, while the red clades stand for maximum bootstrap values, but all those <50% are not marked.

To investigate the origin of the bZIP family members, some known bZIP sequences were used as queries for BLASTP (i.e., protein blast) in the non-redundant protein sequences database and for the HMMER (i.e., Hidden Markov Model) search. As shown in Figure 1a, distinct numbers of bZIP proteins were selected from 23 representative species, including viruses, bacteria, protozoa and metazoa. These 23 species include *Gallid herpesvirus 2* (Gh2), *Cyprinid herpesvirus 1* (Ch1), *Endozoi- comonas numazuensis* (En), *Endozoicomonas arenosclerae* (or *sp. ab112*) (Ea/Es), *Dictyostelium discoideum* (Dd), *Vitrella brassicaformis* (Vb), *Saccharomyces cerevisiae* (Sc), *Monosiga brevicollis* (Mb), *Capsaspora owczarzaki* (Co), *Trichoplax adhaerens* (Ta), *Amphimedon queenslandica* (Aq), *Nematostella vectensis* (Nv), *Caenorhabditis elegans* (Ce), *Drosophila melanogaster* (Dm), *Helobdella robusta* (Hr), *Octopus bimaculoides* (Ob), *Strongylocentrotus purpuratus* (Sp), *Danio rerio* (Dr), *Xenopus tropicalis* (Xt), *Gallus gallus* (Gg), *Anolis carolinensis* (Ac), *Mus musculus* (Mm), and *Homo sapiens* (Hs). Across these species, a total of 441 of the bZIP proteins were identified, after removal of both incomplete and repeated sequences from the resulting searches from BLASTP and HMMER databases. For the *Gallid herpesvirus 2*, only one bZIP protein (called MEQ) was identified, with a BRLZ domain that has a high sequence identity of 60.82% with the BATF subfamily (Figure S1a). Another homologous protein of Jun was also found in *Cyprinid herpesvirus 1* (with accession No. YP_007003813 in GenBank), with an 87.5% BRLZ sequence consistency with human Jun (Figure S1b). More interestingly, additional two bZIP proteins were found in these two marine bacteria strains *E*. *sp. ab112* and *E. numazuensi*, which are designated Nach1 and Nach2, respectively, based on its high homology with the known CNC-bZIP proteins (Figures S2–S4). In metazoans, except vertebrates, the number of bZIP proteins were approximately between 12 and 19 (Figure 1a), for example, sea urchin (*Strongylocentrotus purpuratus*) up to 19 proteins. The more bZIP proteins were identified in vertebrates, e.g., the human (*Homo sapiens*) up to 53 bZIP proteins, six of which belong to the CNC-bZIP subfamily.

The sequences of the essential bZIP domains within the 441 proteins were extracted using the SMART software, and the conservative principle is presented on the basis of the MEME analysis. As illustrated in Figure 1b, the conservative domain of about 60 aa in length mainly includes the BR and adjacent LZ regions. The bipartite NLS-containing BR region consists of about 21 aa with the conserved motif -K/R-X3-(R/K)2-X-K/R-N-R/K/N-X-A/S/Y-A/V-X2-C/S-R-X-(K/R)3- (in which X indicates any amino acid residues). Of note, the -C/S-R- peptide exists in a majority of bZIP proteins, but is replaced by -A-R- in the viral MEQ and bacterial Nach1 (Figures S1 and S2) or by -Y/F-R in the yeast activator proteins (Yaps, Figure S5). The BR-adjacent LZ region is composed of six rounds of the conserved heptad repeats, i.e., wheeled by seven residues (denoted *a* to *g*), in which the typical residues at two positions “*a*” and “*d*” are key in forming a hydrophobic interface and essential for the dimerization of related bZIP proteins. Within the LZ regions, almost all the “*d*” positions are highly conserved and occupied primarily by leucine (L) or other hydrophobic residues. The third “*a*” position asterisked is also highly conserved by asparagine (N), while glutamic (E) at the first to fourth ‘*g*’ positions are relatively conserved (Figure 1b).

To further clarify the phylogenetic relationships of the selected 441 bZIP proteins by analyzing the sequence conservation of their BRLZ domains, we constructed the neighbor-joining phylogenetic tree with 17 distinct clades (Figure 1c). Notably, the unicellular yeast Yap proteins, along with *Hs*-CHOP (CCAAT/enhancer-binding protein (C/EBP)-homologous protein), *Mm*-CHOP, *Dr*-CHOP and *Xt*-CHOP, were collectively gathered into a branch Yap subfamily, by employing the well- supported bootstrap values. The remaining 430 BRLZ sequences were clustered into additional 16 branches, each of which has a potential individual difference from others, within their mutual relatively independent and interrelate connective evolutionary trajectories (Figure 1c). In the clockwise direction, the gap of the phylogenetic tree serves as a starting point, followed by the scenario of phylogenetic tree with distinct branches that were separately clustered as XBP1 (X-box binding protein 1), CNC, sMaf (small musculoaponeurotic fibrosarcoma oncogene homolog), Maf, BATF, CREB, ATF6, OASIS (old astrocyte specifically-induced substance, also called CREBP3-like protein 1), PAR (proline- and acid-rich bZIP), E4BP4 (E4 promotor-binding protein 4), ATF4, ATF3, Fos, ATF2, Jun, C/EBP, subfamilies (of which all their BRLZ sequences were also aligned, as shown in supplemental Figures S3, S4, and S6–S13).

Notably, *Mb*-ATF4L is the more primitive homologue among the ATF4 subfamily, which together with the ATF3 and Fos subfamilies appears to originate from a big predecessor branch (Figure 1c and Figure S9b). The XBP1 subgroup with a high conservation of yeast HAC1 was clustered independently, but closely related to the Yap group. Both CNC and Maf (that is combined with sMaf) subfamilies shared a common evolutionary branch (which seemed to share a high sequence conservation with *Co*_XP_004343898, in Figure 1c and Figure S1d). Of note, all Nachs were clustered into the CNC clade. Four subfamilies of BATF, CREB, ATF6, and OASIS were gathered together, but the latter two subfamilies still retain a type-II transmembrane (TM) region, respectively. The ATF2, Jun, and C/EBP subfamily shared a large clade, while the E4BP4/ NFIL3 and PAR subfamilies were also clustered into another big clade (Figure 1c). In addition, it should be noted that the bZIP proteins, labeled by red stars, denote those not yet identified in the past, and others labeled by blue stars stand for ambiguously classified bZIP proteins. For example, the former representative is *Vb*-bZIP-TF2/3 in the XBP1 clade, whilst the latter representative is *Ch1*-(YP_007003813) in the Jun subgroup.

More interestingly, the BATF and Jun subfamilies (including other AP-1 family members Fos and ATF2) are inferable to be originated from the putative earliest primogenitor existing in the viruses (Figure S1a,b), whereas both the C/EBP (Figure S1c) and CNC (Figures S2–S4) subfamilies appear to be stemmed from the marine bacteria, albeit relevant details of their early evolutionary mechanisms are unknown. Surprisingly, all other nine subfamilies Maf/sMaf, CREB, OASIS/ATF6, ATF2, ATF4, FOS, and PAR have shared with those derivatives from the putative primogenitor- originated protozoans possibly selected before the dawn of metazoans (Figures S6–S13). In addition, there exists a commonly sharing predecessor of yeast Yap proteins with metazoan CHOP subgroups, closely related with the XBP1 subfamily.

### 2.2. A Novel Evolutionary Branch of the CNC-bZIP Subfamily from Ancestral Nach Proteins

**Figure 2:**
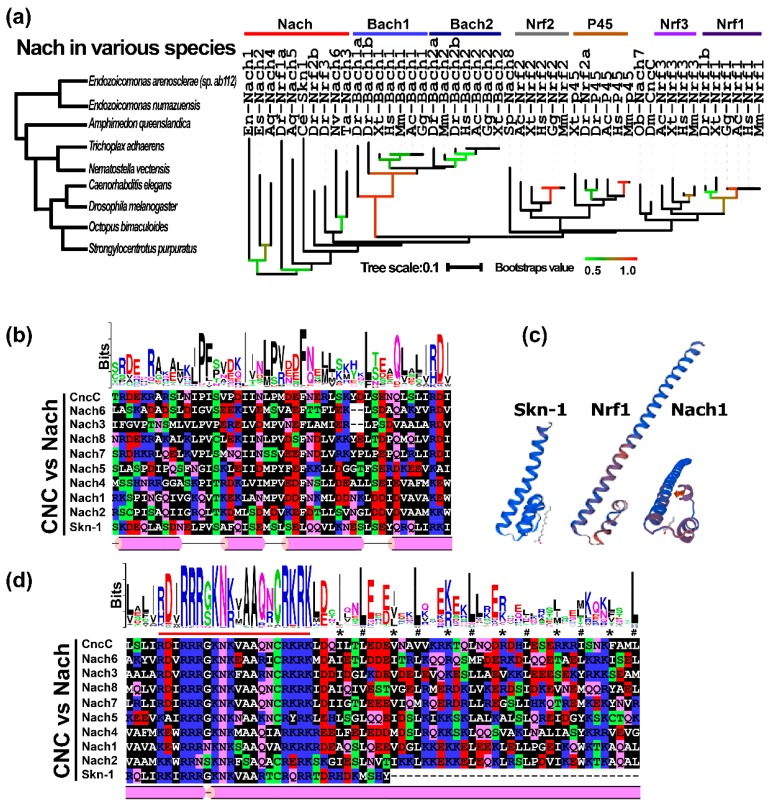
Phylogenetic analysis and sequence structure of the CNC-bZIP proteins in various taxas. (**a**) Left panel shows the putative evolution of distinct organisms with a novel subgroup Nach, which is located in the right smaller neighbor-joining (NJ) phylogenetic tree that was generated by using the MEGA 6.0-based analysis of the full-length Nach/CNC-bZIP proteins across 15 different species with 1000 bootstrap replicates. Multiple sequence alignments of both CNC (**b**) and BRLZ (**d**) domains with distinct characteristics were analyzed by using different tools DNAMAN8.0, PSIPRED, MEME and Web-logo (obtained from all Nach/CNC-bZIP proteins) with distinct default parameters. The red line represents the nuclear localization signal (NLS) in the basic region, while the symbols * and # represent the “*a*” and “*d*” positions in heptad repeats of LZ region, respectively. Similar secondary (**b**,**d** on the bottoms) and tertiary (**c**) structures of the CNC-BRLZ domains within Nrf1 and Nach1 were modeled by using the SWISS-MODEL tool, based on the temple of known homological domain structure of Skn-1.

According to the current literature as far as we know, the CNC subfamily of bZIP transcription factors is composed of NF-E2 p45, Nrf1, Nrf2, Nrf3, and their transcriptional repressors Bach1 and Bach2 in vertebrates, in addition to both Cnc and Skn-1 proteins found in *Drosophila* and *Nematodes*, respectively [26]. To further investigate the evolutionary origin of these CNC-bZIP proteins, we herein used CNC-bZIP transcription factors from humans as inquiry sequences for BLASTP (blast protein) from non-redundant protein sequences database and for HMMER search. As shown in Figure 2a, it was discovered that, apart from Skn-1 and Cnc in *Caenorhabditis elegans* and *Drosophila melanogaster*, the original homologues with the CNC-bZIP proteins are, indeed, objectively present in the marine bacteria *E.* sp. ab112 (Nach1) and *E. numazuensis* (Nach2). Besides Nach1 and Nach2, other orthologues with CNC-bZIP were further searched in the multicellular organisms including *Amphimedon queeslandica* (Nach4/5), *Trichoplax adhaerens* (Nach3), *Nematostella vectensis* (Nach6), *Octopus bimaculoides* (Nach7), and *Strongylocentrotus purpuratus* (Nach8). Hence, these Nrf and CNC homologues 1 to 8, according to the evolutionary status from origin to advance of the distributed species (on the left panel of Figure 2a), were named as Nach1 to Nach8, respectively (Figure 2a, right panel). In addition, it should be noted that among vertebrates, *Danio rerio* (zebrafish) has also given rise to 10 CNC-bZIP homologues, but only four of CNC-bZIP proteins exist in *Gallus gallus* (chicken) with a constructive loss of NF-E2 p45 and Nrf3 from within the genome.

To gain in-depth insights into the phylogenetic relationship of these CNC-bZIP proteins, with their full-length amino acid sequences, all 48 identified CNC-bZIP factors were allowed for building a small neighbor-joining phylogenetic tree. As shown in Figure 2a (right panel), most of the Nrf1 and Nrf3 subgroups were clustered into a big branch, sharing with both *Dm*_Cnc and *Ob*_Nach7. In contrast, a parallel clade comprised mostly of two subgroups of Nrf2 and p45 proteins. All these Nrf proteins were gathered together and also shared a last common progenitor with *Sp*_Nach8. The progenitor was also likely situated at a similar generation to another progenitor shared by between Bach1 and Bach2. These two progenitors were inferable to be originated from the hierarchical root comprising mostly Nach proteins. These putative ancestral Nach proteins retain a high evolutionary conservation with zebrafish Nrf1a, Nrf2b and Nrf3, in addition to Skn-1 of Nematodes (that lacks the fundamental LZ region) (on the right panel of Figure 2a).

The multiple sequence alignment of the above-identified CNC-bZIP proteins revealed one of the most conserved motifs, -φ^10^-ϕ-I/L-P/Q^13^-F/φ-X2-ϕ2-I/L-φ/T^20^-ϕ-L/M-P/S^23^-V/R^24^-ϕ-D/E-F-N/Q-X-φ2-X4-L/F-X3-Q/ϕ-φ-X-φ-φ^44^- (in which φ and ϕ represent any hydrophobic and hydrophilic aa residues respectively, besides X denoting any aa) within their CNC domains (Figure 2b and Figure S3). Notably, a remarkable difference between the Bach1 and Bach2 subgroups appears to be made by the latter 20th position-specific threonines (*T*, in Bach2) or the former 23rd position-specific serines (*S*, in Bach1). In addition to their 24th position-specific arginines in both subgroups, which are distinctive from any hydrophobic residues occupying at these same corresponding positions in all other CNC-bZIP subgroups. Overall, the motif is highly conserved by its sequence identity with equivalents existing among those advanced eukaryotes from octopus to humans, but in some lower lineages, it is a relatively less conservative across all their CNC domains. For example, glutamine (Q) is occupied specifically at the 13th position of the bacterial Nach1 and Nach2, but is replaced by proline (P) within almost all other CNC-bZIP proteins (Figure S3). Furthermore, the secondary structure of this domain is folded into three α-helixes and the N-terminal part of the fourth α-helix, all of which were separated by linear coils (Figure 2b,c).

Similarly, their BRLZ domains also contain the typical basic region and leucine zipper within all CNC-bZIP proteins (Figure 2d). There are highly conserved 21-aa residues -B-D/E-φ-R3-G/S-K-N-K/R-φ-A2-Q/R-N/K-C-R-K-R-K-φ- (where B indicates a basic residue) in the basic-region. Moreover, the leucine zipper region is composed of six heptad repeats of leucines at the “*d*” positions in the α-helical coiled coil as wheeled (Figure 2c,d), in which the first to the third repeated leucine residues (and also the last one) is highly consistent in all CNC-bZIP proteins except Skn-1 (lacking this LZ domain), but the remaining fourth and fifth repeats are less conservative (also see Figure S4). In addition, the homology modeling of the CNC and adjacent BRLZ domains from Skn-1, Nrf1, and Nach1 by the SWISS-MODEL tool have predicted that the latter two proteins have a similar three-dimensional structure to the known template of Skn-1 and other bZIP proteins (Figure 2c).

### 2.3. Distinct Subgroups of the Membrane-Bound bZIP Transcription Factors

**Figure 3:**
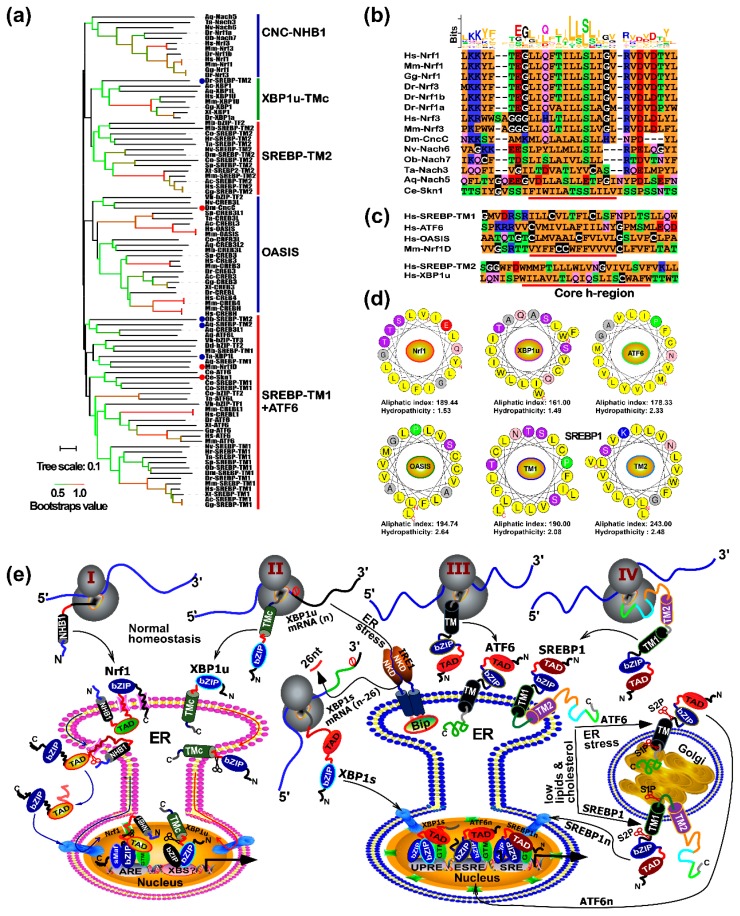
Classification of the current known TM-containing transcription factors. (**a**) All the putative TM-containing domains within bZIP and SREBP proteins were subjected to construction of the neighbor-joining phylogenetic tree by the MEGA 6.0 analysis with 1000 bootstrap replicates; Respectively, the red and blue dots represent CNC-NHB1 and others which of clustering is not gregarious. (**b**) A multiple sequence alignment, with a color Logo of those NHB1-associated TM regions within related Nach/CNC-bZIP proteins, was carried out by using the DNAMAN8.0 and MEME tools. (**c**) Shows two similar sequence alignments of additional TM domains of ATF6, OASIS, Nrf1D and XBP1u with SREBP1-TM1 and -TM2, of which the hydrophobic h-region cores are underlined. (**d**) Six wheels of a-helixes are folded by the NHB1-associated TM1 of Nrf1, the C-terminal TM region of XBP1u, the central TM domains of ATF6 and OASIS, as well as SREBP1-TM1 and -TM2, respectively. Both aliphatic index and hydropathicity were also calculated. (**e**) Four distinct membrane-topobiology models are proposed to give a clear explanation of these TM-containing transcription factors. With distinct catalogues of dynamic topological folding within and around the ER and/or Golgi apparatus, before being translocated out of membranes in order to be released and transferred into the nucleus, prior to activating different sets of cognate target genes, under normal homeostatic or the ER-derived stress conditions.

As stated above, we have analyzed the evolution of the CNC-bZIP family based on the BRLZ domains. Further, given that this family has another unique feature, which enables Nrf1, Nrf3, CncC, and Skn-1 to be anchored within and around the endoplasmic reticulum (ER) membrane through the N-terminal homology box 1 (NHB1) peptides [27,28], however, the origin of such TM-associated NHB1 peptides remains unknown. To explore this, we thus employed the TMpred and TMHMM tools to predict almost potential TM peptides within identified bZIP proteins. The results showed that the putative TM domains are also present in ATF6, OASIS, XBP1, and other ambiguous bZIP proteins, besides most of the CNC/Nach subgroup (Figure 3a). In addition, a slight far homologous subfamily of the sterol-regulatory element binding proteins (SREBPs) is well-documented as the TM-bound basic helix-loop-helix zipper (bHLH-ZIP) transcription factors, which contain two distinct TM domains, denoted SREBP-TM1 and -TM2 from its N-terminal to its C-terminal ends, respectively. Such these putative TM sequences were manually used for further analyses of both their hydrophobicity and conservation. The resulting phylogenetic tree with several branches is constructively extended to distinct five major clades (as illustrated in Figure 3a). Notably, most of the CNC-NHB1 subfamily (in which NHB1 is found in Nach3, 5, 6, and 7, besides Nrf1 and Nrf3 as shown in Figure 3b) appears to be gathered individually, but shares a *de facto* common clade with two close small subgroups of XBP1-TMc and SREBP-TM2 (Figure 3a, upper). An exception occurred, while the NHB1 peptides of CNC and Skn-1 were, respectively, included in an individual OASIS subfamily and another big class of the SREBP-TM1 combined with the majority of ATF6 subfamily members; all these TM regions were further clustered together into a large clade (Figure 3a).

According to the current membrane-topological knowledge [29,30], it is plausible that the *bona fide* TM domains are dictated by their constitutive core hydrophobic (h)-regions spanning across membranes, while distinct orientations of these segments within membranes are predominantly determined by the charge differences along and between its n-region and c-region flanking the core h-region. Therefore, we further aligned multiple sequences of putative TM domains in the CNC-NHB1 subgroup including Nach proteins (Figure 3b), the C-terminal TM domain of mouse Nrf1D (i.e., Nrf1D-TMc, that is distinctive from the NHB1 peptide of prototypic Nrf1), human SREBP-TM1, SREBP-TM2, and others from human ATF6, OASIS, and XBP1u (Figure 3c). The results revealed that they are composed of the major hydrophobic residues through their core h-regions. In particular, almost identical sequences of the core TM h-regions were presented in the entire vertebrate CNC-NHB1 subgroup (Figure 3b). Nevertheless, the less conservative TM domains were also observed in each of the four novel Nach3, 5, 6, and 7 proteins. Furthermore, although the C-terminal TM regions from both mouse Nrf1D and human XBP1u share a certain structural conservation with the human SREBP-TM2 domain, they do only share quite a poor consistency within their sequences as aligned (Figure 3c). By sharp contrast, an additional highly consistency is provided by SREBP-TM1 and homologies from the majority of both OASIS and ATF6 subgroups (upper panel).

Subsequently, to give a clear explanation of topological folding of these membrane-proteins, such typical TM α-helixes of Nrf1 (as a major representative of the CNC-NHB1 subgroup), XBP1u, ATF6, OASIS, as well as SREBP-TM1 and -TM2 were wheeled by the HeliQuest tool, with their aliphatic indexes and hydropathicity estimated (Figure 3d). These six α-helixes are endowed with highly estimated values, of which SREBP-TM2 possesses the highest aliphatic index up to 243, while the highest hydropathicity of OASIS’ TM region is up to 2.64.

The above membrane-bound bZIP and bHLH-ZIP transcription factors were, here, summarily classified into four different categories, which are represented by Nrf1, XBP1u, ATF6, and SREBP1 in order to provide a better understanding of distinct topovectorial processes of these TM-containing proteins integrally folded within and around the ER membranes (Figure 3e). Firstly, the N-terminal SPase-uncleavable NHB1 signal sequence of Nrf1 enables it to be integrally anchored within the ER membranes and determines its topological folding of adjacent domains and their partitioning into the luminal or cytoplasmic sides of membranes (Figure 3e, Model 1) [31]. Subsequently, dynamic repositioning of the luminal-resident transactivation domain (TAD) of this CNC-bZIP factor is driven by p97-fueled retro-translocation pathway into the extra-ER cytoplasmic side of membranes. Its deglycoprotein is therein allowed for the proteolytical processing (i.e., regulated juxtamembrane proteolysis or RJP) by cytosolic proteases to yield a cleaved mature factor. The latter active Nrf1 is released from membranes and translocated into the nucleus, where it enables the formation of a functional heterodimer with its partner sMaf or other bZIP proteins, in order to ensures its different transcriptional regulation of ARE-driven genes [26]. Secondly, the unspliced XBP1u mRNA and its protein are targeted to the ER membrane [32,33,34]. Under normal conditions, the prototypic XBP1u protein is also anchored within the ER membranes through its C-terminal TMc region (Figure 3e, Model 2), in a topology similar to that of the C-terminal Nrf1D, before eliciting its unique function as a transcriptional repressor. Upon the exposure to ER stress, the alternative splicing of XBP1u mRNA by IRE1 to remove its internal 26 nucleotides results in the generation of another open reading frame-shifting variant XBP1s, which lacks an original available TM-targeting peptide, such that XBP1s can directly translocate the nucleus and regulate target genes involved in the ER-to-nuclear unfolded protein response (UPR) (right panel). Thirdly, ATF6 is folded to adapt its initial membrane-topology within and around the ER. When stimulated by ER stress, it will be transported to the Golgi apparatus (Figure 3e, Model 3), in which this protein is allowed for the progressive two-step proteolytic processing by Site-1 and Site-2 proteases (i.e., S1P and S2P) [35,36]. This results in production of a cleaved active factor ATF6n and then activation of its downstream genes driven by ESRE (ER stress response element) or UPRE (UPR element) existing within their promoter regions. Finally, albeit SREBP1 encompasses two TM domains with distinct local topologies integrated within and around the ER, only its TM1 is folded in a similar orientation to that of ATF6. Only when SREBP1-target genes are required for cholesterol and other lipid synthesis, this bHLH-ZIP protein undergoes a similar transfer from the ER through the Golgi to the nucleus, as compared to ATF6 (Figure 3e, Model 4). This topovectorial process of SREBP1 is also attributed to its regulated intramembrane proteolysis (i.e., RIP) by SIP and SIP2, successively, in the Golgi apparatus, so as to generate a cleaved activator SREBP1n before translocating the nucleus [36,37].

### 2.4. Expressive Differentiations of Human bZIP Factors within Their Endogenous Interaction Networks

**Figure 4:**
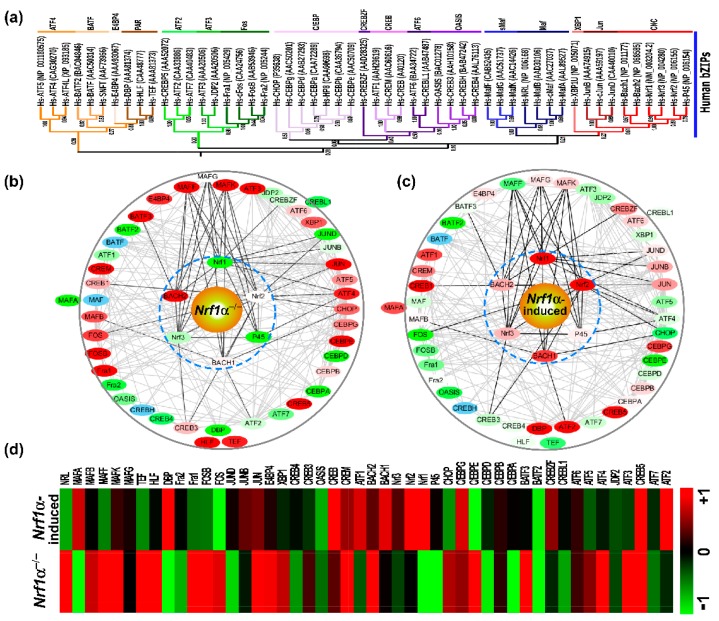

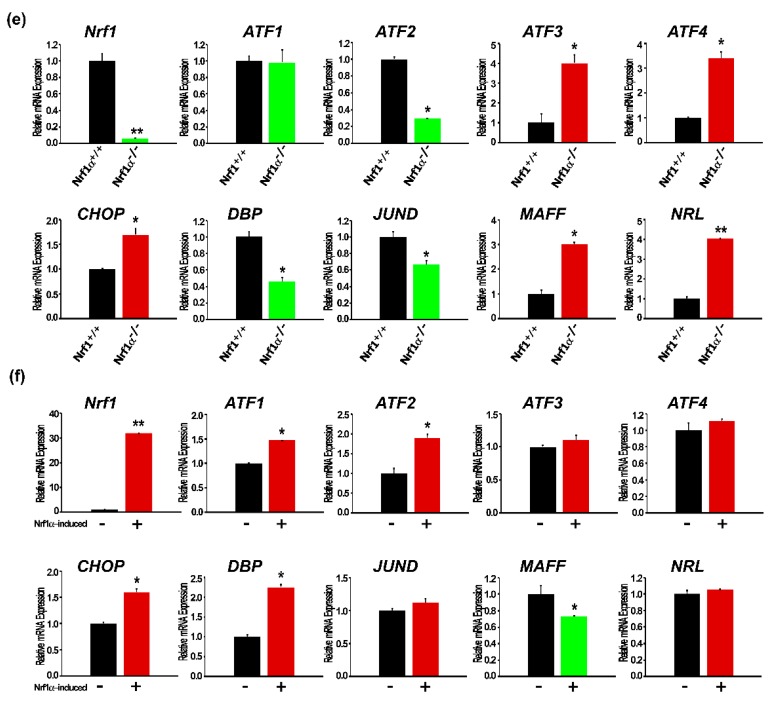
Classification of human bZIP factors within their interaction networks converged on a hub of Nrf1α. (**a**) The phylogenetic tree of 53 bZIP proteins in humans was constructed by the same method as described in Figure 2. (**b**,**c**) Distinct or opposing changes in some nodes within the two interaction networks, which are composed of all human bZIP proteins and also converged on a hub of Nrf1α, were determined following knockout of *Nrf1α* (**b**) or induction of this protein expression by tetracycline treatment of HEK293C^Nrf1^^α^ cells (**c**). Significant up-regulation (Log2-based RPKM value >1) of the indicated genes were red-labeled, whereas down-regulation (Log2-based RPKM value <−1) of other indicated genes were green-labeled. Of note, such a green-to-red gradient of those coding genes demonstrates from being down- to up-regulated. Additional genes without any detectable signals by transcriptome sequencing were also blue-labeled. (**d**) A heat map was made by the Log2-based RPKM values, representing differential expression profiles of human bZIP proteins in *Nrf1α^−/−^* or HEK293C^Nrf1^^α^ (when compared with wild-type *Nrf1^+/+^* HepG2 or un-stimulated HEK293C cells, respectively). Different changes in the expression of some genes were shown to distinct degrees of colors. (**e**,**f**) Relative expression levels of selected bZIP genes were also validated by qRT-PCR analyses of *Nrf1α^−/−^* vs. *Nrf1^+/+^* (***e***), or the stable tetracycline-inducible HEK293C^Nrf1^^α^ vs. un-stimulated cells (**f**). Subsequently, significant decreases or increases (* *p* < 0.05, ** *p* < 0.01) in the expression of some genes were determined. The black histograms indicate the basal *Nrfα* expression (**e**) or its background control levels with no induction (**f**)*.* The red and green histograms show increases and decreases upon knockout of *Nrfα* (**e**) or its inducible expression (**f)**, respectively*.*

In order to gain an in-depth insight into the evolutionary diversity of human bZIP subfamilies during the nature selection, their neighbor-joining phylogenetic tree was further constructed with their full-length amino acid sequences (Figure 4a). The resultant phylogenetic tree displays six major branches including 17 minor subgroups, except for CREBZF (cyclic AMP-response element binding protein zhangfei) clustered individually. Among them, there are high bootstrap values in the nodes between CNC and Jun (0.61) or between ATF3 and Fos (0.98), besides sMaf and Maf belonging to the same large category, while the other three subgroups of ATF6, OASIS, and CREB are also highly homologous. Next, to determine the potential functional differentiation of these human bZIP genes, their mutual interaction networks were established on the solid ground of certain experimentally validated evidence, by employing the STRING program of 51 bZIP-interactive proteins (with a score >0.7 of the moderate confidence) (Figure 4b,c). At the center of the interaction network, the CNC-bZIP subfamily proteins have frequent networking with sMaf, and additional complex links exist between other bZIP proteins.

These two distinct expression profiles of human bZIP factors were obtained by a bioinformatic analysis of the total RNA sequencing datasets. When *Nrf1α* was knocked out by Talens-mediated gene editing in the liver cancer HepG2 cells [38], at least 16 bZIP factors, such as *Bach2*, *MafK*, *MafF*, *Jun*, *FosB*, *Fra1*, *ATF3*, *ATF4*, *NRL*, *HLF*, *TEF*, *CREB5*, *CEBPE*, *E4BP4*, *BATF3*, and *CREM* were up-regulated significantly (with >+1 of the Log2-based RPKM values being calculated) (Figure 4b,d). By contrast, other 7 bZIP factors including *NF-E2 P45*, *MafA*, *JunD*, *DBP*, *CEBPA*, *CEBPD*, and *BATF2* were down-regulated significantly (with <−1 of the Log2-based RPKM values). In addition, *BATF*, *MAF*, and *CREBH* are possibly expressed at much lower levels, so that they were not detected by RNA-sequencing. Conversely, the stable tetracycline-inducible expression of *Nrf1α* in HEK293C^Nrf1α^ cells (Figure 4c,d) caused significant increases in the abundances of *Nrf2*, *ATF2*, *CREB1*, *DBP*, and *CREB5*, while expression of *Fos*, *CEBPE* and *BATF2* was significantly weakened, but both *BATF* and *CREBH* were not detected. These data demonstrate distinct cell-specific expression profiles of certain bZIP genes possibly in different biological contexts.

To ensure that the above RNA sequencing results are reliable, 10 of human bZIP proteins were randomly selected for further examination by real-time quantitative RT-PCR. The results revealed that in *Nrf1α^−/−^* cells, *ATF3*, *ATF4*, *CHOP*, *MafF*, and *NRL* were significantly up-regulated at their mRNA levels (*p* < 0.05), whereas *Nrf1*, *ATF2*, *DBP*, and *JunD* were markedly down-regulated (*p* < 0.05), but expression of *ATF1* was unaltered (Figure 4e). These are consistent with the RNA-seq data. By sharp contrast, induction of *Nrf1α* by tetracycline treatment of HEK293C^Nrf1α^ cells resulted in significant increases (*p* < 0.05) in the inducible expression of *Nrf1*, *ATF1*, *ATF2*, *DBP*, and *CHOP* (Figure 4f), but only caused a marked down-regulation of *MafF* alone (*p* < 0.05), in addition to no obvious changes in the expression of *ATF3*, *ATF4*, *JunD*, and *NRL*. These collective results of all other genes, except for *CHOP*, are in accordance with the RNA-seq data.

### 2.5. Nach1 Shares Conserved Domains with Other CNC-bZIP Factors at Differently Regulating Target Genes

**Figure 5:**
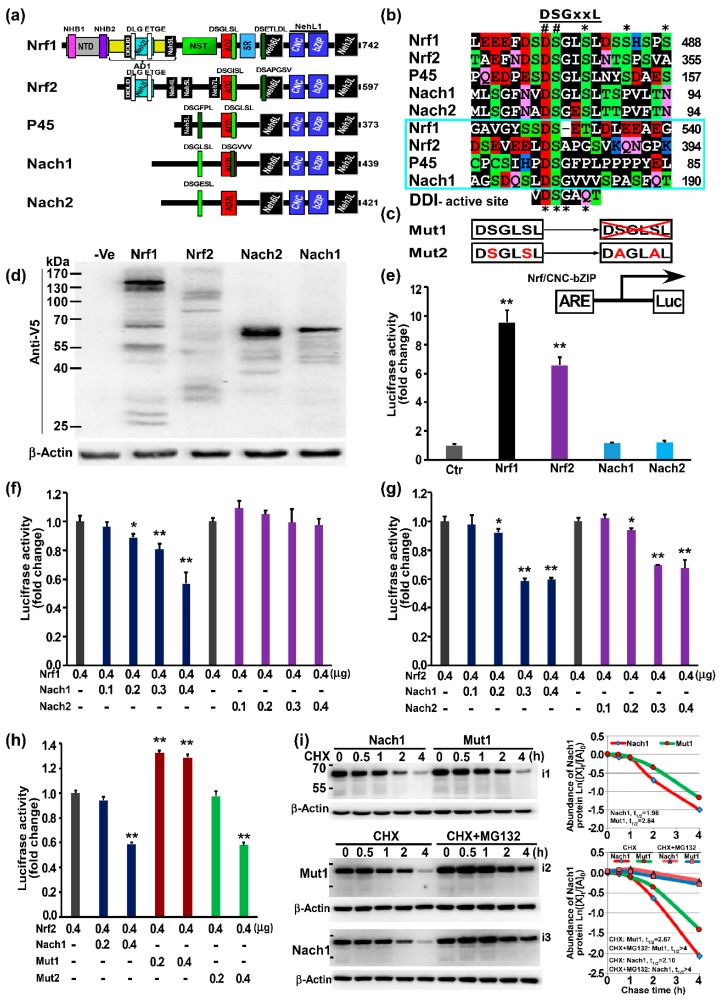
Distinctions in the regulation of ARE-driven reporter gene activity by Nrf1 and Nrf2 from Nach1 and Nach2. (**a**) Schematic representation of structural domains of Nrf1, Nrf2, NF-E2 p45, Nach1 and Nach2, in which the locations of a canonical DSGxSL degron and another non-canonical DSGxxL motif are indicated. (**b**) A multiple alignment of the DSGxSL and DSGxxL (boxed)-adjoining sequence within Nrf1, Nrf2, NF-E2 p45, Nach1 and Nach2. Two similar, but different, degron motifs are highly conserved with the enzymatic active sites (DSGxQx) of the DDI aspartic proteases and hence denoted as a putative suicidon herein. Of note, the critical identical residues DS are indicated by symbols (#), while the consensus GSK-3β phosphorylation sites are marked by another symbols (*). (**c**) Diagrammatic representation of the DSGLSL motif and its mutants of within Nach1; and (**d**) western blotting of HepG2 cells that had been transfected with expression constructs for C-terminally V5-tagged Nrf1, Nrf2, Nach1 or Nach2, while β-Actin served as an internal control to verify amounts of proteins that were loaded in each well occasion. (**e**) ARE-driven luciferase reporter gene activity was measured in HepG2 cells. Respectively, they were transfected for 24 h with expression constructs (0.4 μg of cDNA) for Nrf1, Nrf2, Nach1 and Nach2, together with *GSTA2- 6×ARE-Luc* plasmid (0.2 μg) and pRL-TK (0.1 μg, as an internal control)**,** and allowed for a 24-h recovery from transfection before being disrupted in the lysis buffer. The data were calculated as a fold change (mean ± SD) of transactivation by the indicated Nach/CNC-bZIP factors. Significant increases (* *p* < 0.05; ** *p* < 0.01) in the transactivation activity of ARE-driven reporter gene are determined relatively to control values. (**f**,**g**) Additional two measurements of ARE-driven luciferase reporter gene activity were carried out in HepG2 cells. They were transfected with an expression constructs (0.4 μg of cDNA) for Nrf1 (**f**) or Nrf2 (**g**) in a combination with distinct concentrations (from 0.1 to 0.4 μg) of either Nach1 or Nach2 expression plasmids, together with *GSTA2-6×ARE-Luc* plasmid (0.2 μg) and pRL-TK (0.1 μg) as described above. Significant decreases (* *p* < 0.05; ** *p* < 0.01) in the reporter activity are indicated relatively to controls. (**h**) Similar luciferase reporter activity was also determined in HepG2 cells that had been transfected with 0.4 μg of an expression construct for Nrf2 alone or plus another expression construct (0.2 to 0.4 μg) for Nach1 or its mutants, along with *GSTA2-6×ARE-Luc* plasmid (0.2 μg) and pRL-TK (0.1 μg). The resulting data were calculated as described above. (**i**) HepG2 cells, that had been transfected with an expression construct for Nach1 or Mut1, were treated with CHX (50 µg/mL) alone or plus MG132 (10 µmol/L) for 30 min to 4 h before being disrupted. The relative expression levels of Nach1 or Mut1 in the total lysates were determined by Western blotting with ant-V5 antibody (left panels). The intensity of indicated proteins with distinct half-lives was quantified and shown graphically (right panels).

For an in-depth insight into functional domains of Nach1 and Nach2 required for the regulation of ARE-driven genes (as evidenced by its orthologues of the CNC-bZIP proteins Nrf1, Nrf2, NF-E2 p45) [26], their conserved structural domains were also presented schematically (Figure 5a). The schematic shows that the bacterial Nach1 shares similar structural domains closer to those of human NF-E2 p45 than Nrf1 and Nrf2. Thus, we assumed that p45 is preserved as an intermediate possibly originated from the bacterial Nach1. However, Nach1 lacks the Neh5L domain (Figure 5a and Figure S2B), which is essential for transactivation of ARE-driven genes by all the known CNC-bZIP activators [31,39,40]. Thereby, we postulated that Nach1 could act as a transcriptional repressor as Bach1; this is based on a considerable sequence consistency of between both BRLZ domains (Figure S13). The putative negative function of Nach1 was further inferable to be monitored possibly by its potential degron DSGxSL (i.e., canonical) and/or another similar motif DSGxxL (i.e., non-canonical), both of which have distinct locations from equivalents of NF-E2 p45, Nrf1, and Nrf2 (Figure 5a and Figure S2g), while Nach2 only retains the canonical DSGxSL degron in a similar location to that of Nach1.

To determine the biological function of Nach1 and Nach2, HepG2 cells were transfected with each of expression constructs for Nach or CNC-bZIP protein, of which C-terminal ends are tagged by the V5 peptides. Western blotting revealed that either Nach1 or Nach2 was expressed as a major protein of 65-kDa estimated on 10% PAGE gels (Figure 5d), which was accompanied by a ladder comprising of several degraded polypeptides between 65-kDa and 30-kDa. This indicates that both proteins are unstable and rapidly degraded. Further, luciferase assay showed that over-expression of Nrf1 or Nrf2 significantly increased ARE-driven reporter gene activity in HepG2 cells; they were activated to 9.56 and 6.60 folds of the background level. However, over-expression of Nach1 or Nach2 had no significant effect on activity of ARE-driven reporter gene (Figure 5e). Subsequently, co-transfection experiments showed that Nach1, but not Nach2, caused a marked decrease in the Nrf1-mediated transactivation activity in a dose-dependent manner (Figure 5e). Similarly, another dose-dependent inhibitory effect of Nach1 and Nach2 was also, respectively, exerted on the Nrf2- mediated transactivation activity of ARE-driven reporter (Figure 5e,f).

Next, to further identify a role of the putative DSGxSL degron within Nach1, its mutant 1 (i.e., Mut1, which was yielded by mutagenesis to delete the entire DSGLSL motif from Nach1) and Mut2 (in which the DSGLSL motif was mutated to DAGLAL) were subjected to co-expression with Nrf2 in ARE-driven reporter assays (Figure 5c,h). As anticipated, Mut1 rather than Mut2 resulted in a striking de-repression of Nrf2, so that Nrf2-mediated reporter gene activity appeared to be rescued significantly to 1.41~2.19-fold transactivation (Figure 5h). 

Further pulse-chase experiments of HepG2 cells that had treated with cycloheximide (CHX, that inhibits biosynthesis of nascent proteins) alone or in combination with the proteasome inhibitor MG132 revealed that Mut1 caused an obvious increase. When compared with wild-type Nach1 protein having a shorter half-life defined to be 1.98 h (=119 min), the Mut1 protein was more stable because its half-life was extended to be 2.84 h (=170 min) following CHX treatment (Figure 5i, i1 and upper graph). However, the turnover of both Nach1 and Mut1 proteins was still prolonged by MG132, with similar half-lives determined to be over 4 h after treatment of cells (i2, i3 and lower graph, and also see the whole gel images in Figure S14).

## 3. Discussion

### 3.1. A Phylogenetic Web of the bZIP Transcription Factors

During the evolution by variation and selection, the diversity of living organisms increases their biological complexity of distinct species to survive in a changing environment [41]. To meet the needs of normal homeostatic development and growth, as well as the biological response for patho-physiological adaptation and cytoprotection against stress, distinct transcription factors have been selected to regulate expression of different target genes during evolution [20]. In fact, it is found here that disparate species lineages are represented by divergent distribution of bZIP transcription factors existing in 14 representative metazoans, 5 typical protozoans, 2 bacteria, and 2 viruses. The number of bZIP proteins increases with increasing morphological and behavioral complexities in distinct vertebrates (e.g., 48 and 53 of bZIP proteins have been identified in NLS to human, respectively). However, the number of orthologues in protozoa, including choanoflagellates and fungi, is relatively less, because there are eight bZIP families (Figure 1a). Thus it is deduced that the first round of putative expansion and diversification occurred in protozoan lineages, whereas the second round of incremental expansion and diversification occurred in metazoan lineages except for vertebrates. Gradually, in vertebrates, the gene number is determined by maximal expansion and diversification to certain extents. Taken together, these demonstrate that the evolution of distinct eukaryote species is positively correlated with expansion and diversification of the bZIP superfamily. Throughout the metazoan evolutionary process, all their bZIP proteins are conserved at certain extents, albeit many of both their orthologues and paralogues have been endowed with strikingly different interactive specificities as described in Reference [42]. This notion is further supported by bioinformatic analyses of the consensus BRLZ domains from distinct bZIP subfamilies (Figures S4–S13), albeit their evolutionary conservation was not elucidated by an early study of six main eukaryotic lineages, including Holozoa, Fungi, Amoebozoa, Plantae, Heterokonta, and Excavata [23]. 

Within all distinct subfamilies of bZIP proteins, an essential conservative domain is composed of both BR and LZ regions (Figure 1 and Figures S4–S13). The dimeric specificity and stability of bZIP proteins are dictated principally by leucine and other hydrophobic residues occupied dominantly at these two, “*d*” and “*a*” positions of heptad repeats wheeled, respectively [43,44]. When charged residues are placed at the “*a*” position, they are conferred to drive heterodimerization of bZIPs in *Arabidopsis thaliana*, whereas the asparagine residue at the “*a*” position develops a tendency to form a homodimer [45]. Here, we further observed that besides the leucine residues at the last “*d*” position preserved in the four subfamilies of CNC, Maf, ATF6, and OASIS, the histidine residues at this position are also conserved in another five subfamilies of Fos, Jun, ATF2, ATF3, and BATF, but the poor conservation occurs in other subfamilies. This implies the last “*d*” position is also responsible for distinct dimerization. Additional potential differences among distinct bZIP subfamilies are also postulated to determine their dimeric stability. This is evidenced by the finding that the asparagine residue at the third “*a*” position is highly conserved in most bZIP proteins, because it can also elicit a limitation of LZ dimerization [46,47]. 

It is necessary to gradually trace potential divergence of the bZIP superfamily, through further evolutionary analysis of all these clusters of their BRLZ domains into 17 clades (Figure 1), including 16 typical subfamilies that were subjected to the coiled-coil arrays in humans [44], except for an extra classification in yeast Yaps. Notably, an amino acid sequence consistency of 72.22% between BRLZ domains of human C/EBP and its homologous protein from marine bacteria (with the GenBank accession No. WP_062270874) is determined (Figure S1c); both also share an evolutionary stem with Skn-1 (as a member of the CNC-bZIP subfamily) (Figure 1c and Figure S15). Two additional orthologues (i.e., Nach1 and Nach2) of the CNC-bZIP subfamily have emerged in the aforementioned marine bacteria (that are Gram-negative, aerobic and motile, belonging phylogenetically to the class of *g*-proteobacteria [48]). The bacteria were isolated from marine sponges *Arenosclera brasiliensis*, which still retain Nach4, Nach5, and other 15 bZIP factors (Figure 2 and Figures S3–S5). Notably, the Nrf/CNC subfamily also shares a common ancestor with human Jun (Figure 4a). 

Interestingly, two homologous proteins of both the Jun and BATF subfamilies, with 87.5% and 60.8% amino acid sequence consistency of their BRLZ domains, have existed in *Cyprinid herpesvirus 1* and *Gallid herpesvirus 2*, respectively (Figure 1c and Figure S1). They are also highly conserved with Fos, ATF2 and other homologues (Figures S1b and S15). In addition, a small variant bZIP factor encoded by the human T-cell leukemia virus type1 (HTLV-1) (i.e., HBZ, acting as a transcription repressor of viral replication and proliferation) [49,50] was redefined during evolution to retain double the BR region and a unique LZ region, which comprises seven rounds of heptad repeats and shares a highly homology with MEQ, Nach1, Nach2, p45, and Nrf1γ (Figure S2f). Collectively, it is thus postulated that there exists a potential common far-reaching origin of bZIP proteins during *a priori* programmed evolution. This could be identifiable from the viruses (e.g., *herpesvirus*, [51,52]) to the prokaryotes (e.g., marine bacteria), which have continually evolved into distinct species of eukaryotes [53,54], although it remains unknown about the details of the early evolutionary mechanisms whereby the putative common ancestral bZIP gene has been transferred to its hosts. 

Apart from the variant HBZ from the retrovirus HTLV-1, the identification of a homologous protein of Jun existing in *Cyprinid herpesvirus 1* and another homologous protein of BATF in *Gallid herpesvirus 2*, raises a few of interesting questions. It should be noted that viruses cannot be analyzed in the same manner as the cellular life, because they are polyphyletic (i.e., having many evolutionary origins). Since it is so, there are none of ancestral viral lineages; that is to say, not a single ancestral gene (i.e., a haplotype) has been identified so far for being shared by all viruses [51]. Rather, it is generally considered that viruses reflects the genetics of their hosts (i.e., prokaryotic and eukaryotic organisms), for example, in the case of influenza viruses [55]. The paradigm is further complicated possibly by the wide-spread horizontal gene transfer (HGT, identified as a powerful evolutionary force [56]) from distinct viruses to their hosts. Since the herpesviruses are not of the retrovirus, their bZIP proteins should also not be among those candidate genes for horizontal transfer [56]. This leads to a claim that not a viral bZIP gene is determined to be horizontally acquired in 16 distinct animal genomes, albeit thousands of horizontally-transferred genes including those derived from distinct lineages of viruses and bacteria were identified [57]. 

In a convergent evolutionary event, a retroviral *s*uperantigen *g*ene (*sag*, as a hallmark of viral fossil existing in the betaretrovirus mouse mammary tumor virus (MMTV) and South American herpesviruses infecting monkeys and rats) is horizontally captured by mammalian herpesviruses and integrated into their host genomes [53]. The cross-species transmission of monkey herpesviruses occurred after the acquisition of *sag* twice from separate lineages that are distinctive from MMTV, but it was not reported whether the bZIP genes are horizontally transferred. Intriguingly, additional two bZIP homologous proteins of Skn-1, C/EBP, ATF2, Fos and Jun (Figures S1b and S15) are found to be constitutively expressed in human gammaherpesviruses Epstein-Barr virus (EBV) and Kaposi’s sarcoma-associated herpesvirus (KSHV) [58]. Both bZIP proteins (i.e., BZLF1 and K-bZIP) induce the viral DNA replication in infected lymphoid and epithelial cells, resulting in the development of cancer and autoimmune diseases. Collectively, thus we surmise one quite possibility that after these bZIP proteins were acquired by HGT at certain points of their evolutionary process, they were then optimized and conserved because of a beneficial function for the host, albeit it is unknown about the details of the early events of viral infection in the last common ancestor.

Within the evolutionary “web of life”, the vertical hierarchical bifurcating pathways enable the heritable materials (i.e., genomic DNA) of distinct life forms to be steadily transferred from the parents to off-springs [53]. These pathways are further horizontally linked at distinct hierarchical levels for alternative acquisition of genes between species (e.g., from bacteria to humans). However, the mechanism(s) through which genomic DNA could be transferred among species remains quite unclear [54,59]. Still, it is reasonable to argue that the physical proximity is a prerequisite to promote the potential lateral gene acquisition. Thereby, a kind of the parasitic (i.e., ticks) or viral relationship needs to adequately validate a hypothesis on gene transfer mechanisms. For this point, we cannot, however, establish direct relationship between marine bacteria and humans. As such, the end result revealing that during evolution from marine bacteria to humans, the sequence conservation of Nach and other bacterial bZIP homologues (WP_062270874 and KRG21159) with human equivalents has been clearly established (Figure 2, Figures S1, S3–S5, and S15), but is, by itself, a mystery, and therefore, it is interesting to be further studied.

### 3.2. Nach Is Buded at the Early Evolutionary Stage of the CNC-bZIP Transcription Factors

Interestingly, a subgroup of bZIP transcription factors with a unique conserved CNC domain (Figure 2) comprises NF-E2 p45 subunit, related factors Nrf1 (also called NFE2L1, along with a short form LCR-F1 and a long form TCF11), Nrf2, Nrf3 and the repressors Bach1 and Bach2. Among vertebrates, these CNC-bZIP proteins are highly conserved with their founding member *Drosophila melanogaster* Cnc protein and *Caenorhabditis elegans* Skn-1, but none of their orthologues are identified in plants and fungi [60]. These CNC proteins, except Skn-1, heterodimerize with sMaf [61] or other bZIP proteins such as Jun [62], in order to regulate target genes that are involved in cytoprotection against oxidative and other stresses [63]. However, a limitation that the origin of CNC-bZIP proteins was only traced back to vertebrates presented there, although the first conserved CNC domain was identified in the *CNC* gene product from the *Drosophila melanogaster* [25]. 

Fortunately, a novel subgroup of Nach1-8, with a high homology with all the known CNC-bZIP proteins, are herein identified to be present in the Echinodermata, Mollusca, Actiniaria, Placozoa, Porifera, and bacteria, respectively (Figure 2). This discovery implies that the CNC-bZIP proteins are originated from the marine bacteria to multicellular organism (e.g., *Amphimedon queeslandica*). This is because none of their orthologues have emerged in the unicellular protozoans or other prokaryotes beyond *Endozoicomonas*. Notably, only one or two Nach proteins are found in each of species such as ascidians, sea urchin, octopus, fly, and hydra. Overall, these findings indicate that the expansion and diversification of CNC-bZIP subfamily appear to have occurred only in the vertebrate. Along the early solitary evolutionary branch of the phylogenetic tree is budded by distinct species-specific Nach proteins from the marine bacteria to simple multicellular eukaryotes. This notion is in perfect agreement with an accepted fundamental concept in biology that the eukaryotes (e.g., human) have been evolved originally from the prokaryotes (i.e., marine bacteria), and that their genomic DNA could be transferred among these species by a hierarchic evolutionary process [53,54,59]. For this reason, the sequence evolutionary conservation of all Nach/CNC-bZIP proteins demonstrates that a putative original genomic *Nach* could be transferred and then diversified hierarchically from marine bacteria to humans, but the details of possible vertical and/or horizontal gene transfer mechanisms remain obscure so far. Furthermore, it should be noted that, since a physical proximity is known as a prerequisite to promote possible horizontal gene acquisition, it is inferable that the potential transfer event could take place between the marine bacteria *Endozoicomonas* and its host animals standing aside from humans. This is due to the objective fact that not any one of the *bona fide* orthologuous proteins of the Nach/CNC-bZIP subfamily has emerged in the human-surrounding prokaryotes and viruses (including parasitic and infected ones), albeit these microbiomes could enable a potential physical proximity to marine bacteria and animals. Hence, such possible gene transfer mechanism is an interesting topic to warrant further studies.

The evolution of early unicellular to multicellular organisms is also monitored by biosynthesis of membrane lipids, together with a proper assembly with the key membrane-embedded proteins [53,64]. Nonetheless, it is unknown whether and how a given membrane-associated transcription factor responsible for the lipid synthesis is selected to meet the requirements for this evolutionary process. As a matter of fact, there exists a specific subfamily of membrane-bound bZIP transcription factors, some of which are indispensable for controlling lipid biosynthesis and cellular response. These TM-containing proteins are folded into distinct topologies within and around ER membranes, and then processed into a mature activator in order to be released and translocated into the nucleus before regulating their target genes (Figure 3). In the past two decades, the well-documented transmembrane transcription factors are both the bZIP protein ATF6 and another bHLH-ZIP protein SREBP in mammals; both coordinately monitor expression of key genes responsible for biosynthesis of cholesterol and other lipids to meet the cellular needs [65]. Notably, we have further predicted by using both the TMpred and TMHMM tools, and also summarized all potential TM-containing bZIP proteins across 23 species, which were classified into 4 major subgroups including CNC-NHB1 (also including Nach3, 5, 6, and 7), XBP1u, OASIS/ATF6, and SREBPs. 

In response to ER stress, both ATF6 and SREBP are allowed for a transport from the ER to the Golgi apparatus, in which ATF6 and SREBP-TM1 are enabled for successive proteolytic processing by SIP and S2P to yield their N-terminal releasable portions acting as activators (Figure 3e). As such, a nuance in the processing of both proteins could determine their difference in the ER retention and release signals [66,67]. Among these bZIP proteins, the early presence of the TM domain is found in protozoans, but the TM-bound CNC-bZIP factors appear to be originated from the Actiniaria (sea anemones) rather than bacteria. However, none of similar TM-containing bZIP proteins are searched in either the unicellular organisms or prokaryotes (Figure 3). It is inferable that the TM-bound bZIP protein is likely generated only when a small TM-encoding fragment was probably fused with the non-membranous BRLZ-encoding gene during early evolution. Contrarily, it is also plausible that the absence of TM-containing bZIP transcription factors facilitates to contribute to a simple biology process in the unicellular organisms and prokaryotes. With the increasing complexity of biological behaviors along with distinct evolutionary morphologies, a naturally selected optimal fusion of the TM region with the conserved BRLZ domain is presumed, such as a TM-bound bZIP factor allowing it to be involved in signal transduction, ion transmission, and other life processes. This is further supported by the fact that the TM helices can be conferred on the fusion TM-bZIP proteins to play fatal roles in distinct biological processes [68]. This is also fully consistent with the notion that the TM-containing bZIP players regulate the ER functions through distinct response signaling pathways to defend against the ER-derived stress [69].

### 3.3. Distinct Functions of Nach1 and Nrf1 in Regulating Target Genes

Since the complex relationship between an organismic genotype and phenotype is clearly mediated by many of certain interrelated biochemical networks [70], Metazoans have evolutionarily developed a considerably higher proportion of heterodimeric bZIP interactions to homodimeric ones, along with more network complexity than those generated in the unicellular species [42]. Herein, we have demonstrated that the complex regulatory networks of human bZIP transcription factors, in which the CNC-bZIP factors are closely interactive with sMaf (i.e., MafG, MafF, and MafK) (Figure 4). Further examinations revealed that knockout of either Nrf1α or its constructive induction also enables it to trigger different and even opposing expression profiles of other bZIP genes. These certain genes only need to be active in a particular cell type at any given time, but the transcriptional activity of such genes is finely or quantitatively monitored by upstream bZIP factors, as a functional homo- or hetero-dimer is formed for specifically binding to the genomic DNA motifs, such as AP1- like ARE sequences. These bZIP transcription factors are also often working together to regulate basal and stimulated expression of some key specific genes involved in the responses to various intracellular and extracellular signals, as well as to other stresses from the changing environments. Conversely, the failure of these bZIP factors controlling the activity of given genes ultimately results in the pathogenesis of cancer, diabetes or a wide array of other diseases. The precision expression of their target genes is tightly regulated by the complex regulatory networks of the bZIP superfamily through interacting with their dimeric partners, so as to ensure the adaptive responses to complex and changeable environments, as described by those authors in Reference [71]. Overall, diverse interactions of distinct bZIP transcription factors with different partners elicit different regulatory effects on target genes. In turn, such regulatory effects are also likely monitored by their conserved functional motifs. The regulatory and signal molecules are taken together and form an endogenous network, whose dynamical structures are eventually responsible for health and disease [72].

The present study has demonstrated that the Nach- and CNC-bZIP subfamilies with similar conserved, but slightly different, structural domains (e.g., NTD, Neh2L, Neh5L, NehL1, and Neh3L) and functional motifs (e.g., DLG, ETGE, DIDLID/DLG, and DSGLSL) (Figure 3 and Figure 5, and Figure S1). Further evidence has been provided by us and other groups [73,74,75], revealing that the canonical DSGxSL degron and another similar non-canonical DSGxxL motif within the Nach/CNC factors are involved in the regulation of both their protein stability and transcriptional ability to mediate expression of AP1-like ARE-driven genes, that are responsible for antioxidant, detoxification, and cytoprotection against cellular stress. Since NF-E2 p45 and Nrf3 are subject to their tissue-specific expression in hematopoietic and placental cell lineages, respectively [76,77,78], the transcriptional expression of ARE-driven genes is thus regulated primarily by two master players Nrf1 and Nrf2, essential for maintaining cellular homoeostasis and organ integrity in mammals. The Nrf2 activity is negatively regulated by its DSGISL motif acting as a redox-insensitive β-TrCP (β-transducin repeat- containing protein)-binding degron [73,74]. A similar DSGLSL motif in Nrf1 was also identified as a GSK-3β- mediated phosphodegron targeting this CNC-bZIP protein to the β-TrCP^SCF^-dependent ubiquitin proteasome degradation [75]. More excitingly, the activity of ARE-driven reporter gene mediated by Nrf1 and Nrf2 is significantly suppressed by their homologues Nach1 or Nach2 (both lacking the Neh5L element essential for target gene transactivation).

Since Nach1/2 is *de facto* present in the marine bacteria but not in the human genome, thereby in this human experimental setting, over-expression of Nach1/2 is artificial. This is suggestive of the main difference existing between Nach1/2 and the human Bach1/2, demonstrating that even if both have similar experimental effects as repressors, they rather play diverse roles in regulating distinct subsets of target genes in different species. According to the results (Figure 4b,c) revealing that the CNC-bZIP factors are closely interactive with sMaf subgroup to form a functional heterodimer for binding cognate genes, since Nach1/2 as a newly identified CNC-bZIP member is also subjected to our experimental settings, we speculate a theoretic possibility that they are much likely to interact with a member of the sMaf subgroup, albeit no available evidence has been presented. Nonetheless, it is unfortunate that none of sMaf homologues have been identified so far to exist together with the Nach1/2 factors in the same species of marine bacteria. Therefore, even though Nach1 and Nach2 significantly suppressed the activity of ARE-driven reporter gene mediated by Nrf1 and Nrf2, this suppressive effect should also be only limited to the experimental human HepG2 cells. 

As such, the above suppression is also abolished by a deletion mutant of the canonical DSGxSL from Nach1, but not by another point-mutant DAGxAL (Figure 5). This observation may still imply that the complete degron sequence DSGxSL is available for expression of the reporter examined, but its effect is, indeed, not attributed to these two main serine residues. Further experiments determine that the DSGxSL can also act as a degron targeting Nach1 to the proteasome degradation pathway, but the degradation is not completely prevented by proteasome inhibitors. Intriguingly, both the canonical DSGxSL and non-canonical DSGxxL degrons of related Nach/CNC-bZIP proteins appear to share a certain conservation with the enzymatic active site (DSGxQx) of within the DDI aspartic proteases (Figure 5b). Collectively, it is thus reasonable that these Nach/CNC-bZIP proteins may be auto-destructed by their DSGxSL and/or DSGxxL degrons *per se* (i.e., suicidon designated herein). Such event enables their target genes to be rapidly recovered after their transcription is switched off.

## 4. Materials and Methods

### 4.1. Identification of bZIP Proteins

The BLAST program was conducted to identify bZIP superfamily members with the parameter (*E*-value = *e*^−5^). Meanwhile, the HMM search (http://hmmer.org/) was also employed to identify bZIP proteins with the default parameter (*E*-value = 0.01). The resulting searched sequences were downloaded from the database of NCBI (National Center for Biotechnology), of which the repeated and incomplete sequences were manually removed. In addition, some bZIPs that had been not identified in the past were herein denoted by a nomenclature rule “××_bZIP_TF× (the first double ×× letter is represented by the abbreviation for indicated species’ name, and the last x letter shows the number of unidentified bZIPs in this species.)” (Figure S5). Notably, all the false-positive sequences were removed according to two selection criteria: Firstly, the clan CL0018 from the Pfam database contains three member families (PF00170, PF07716 and PF03131); and secondly, all the BRLZ domains were identified by using the SMART sequence analysis.

### 4.2. Phylogenic Analysis of Structural Domains

For phylogenetic analysis of the BRLZ domains, they were extracted from all the selected bZIP proteins by using a local PERL SCRIPT program, and then aligned using DNAMAN 8.0 (Lynnon Biosoft, San Ramon, CA, USA), T-Coffee Server (Comparative Bioinformatics Group Bioinformatics and Genomics Programme Center for Genomic Regulation (CRG), Barcelona, Spain) and ClustalX 2.0 (Conway Institute UCD Dublin, Dublin, Ireland) with distinct default parameters. The multiple sequence alignments were manually refined and end-trimmed to eliminate the poor scored or divergent regions. Subsequently, the remaining unambiguously aligned sequences were subjected to construction of the neighbor-joining phylogenetic trees, by using the MEGA version 6.0 (Tokyo Metropolitan University, Tokyo, Japan) (with a gap treatment: partial deletion; model of evolution: the Poisson model; 1000 bootstrap replications), which are displayed by the iTOL program [79]. Moreover, the conserved motifs of CNC and BRLZ domains were also analyzed by the MEME and Web-logo tools with default parameters. In addition, the secondary structures of CNC and adjacent BRLZ domains were predicted by using the PSIPRED tool, and the 3D structures of Nrf1 and Nach1 with the homology of Skn-1 were further modeled by the SWISS-MODEL software (University of Basel, Basel, Switzerland).

### 4.3. Bioinformatic Analysis of TM-Containing Transcription Factors

The above-selected bZIP proteins were used to predict TM-containing transcription factors by TMpred (EMBnet node, Lausanne, Switzerland) and TMHMM Tools (Technical University of Denmark, Copenhagen, Denmark). The identified TM domains were subjected to the phylogenetic analysis, with multiple sequence alignment, and conservative analysis. In addition, six TM-folded α-helix properties of Nrf1, XBP1u, ATF6, OASIS, SREBP-TM1, and -TM2 were calculated with the HeliQuest tool (Université de Nice Sophia Antipolis and CNRS, Valbonne, France).

### 4.4. Interaction Network and Transcriptomic Analysis

The interaction networks of between bZIP proteins in humans were also constructed with the STRING software (as a Search Tool for the Retrieval of INteracting Genes and/or proteins, http://string-db.org/) [80]. Relative levels of gene expression were calculated as RPKM (Reads Per Kilobase per Million mapped reads). According to the Log2-based RPKM value, the heat map was also generated with the MEV4.9 program (Dana-Farber Cancer Institute, Boston, MA, USA).

### 4.5. Experimental Cell Lines

Experimental cell lines, including human hepatocellular carcinoma HepG2 (i.e., *Nrf1^+/+^*), *Nrf1α^−/−^* (established by Talens-mediated *Nrf1α*-specific knockout in HepG2), human embryonic kidney (HEK293) and HEK293C^Nrf1α^ (with stable tetracycline-inducible expression of Nrf1α established in human embryonic kidney) were cultured in a 37 °C incubator with 5% carbon dioxide, and allowed for growth in Dulbecco’s modified Eagle’s medium (DMEM) with 25 mmol/L high glucose, 10% (*v*/*v*) fetal bovine serum (FBS), 100 units/mL penicillin-streptomycin.

### 4.6. Validation of Gene Expression by qRT-PCR

Total RNAs was extracted from cell samples, by using an RNA extraction kit (TIANGEN, Beijing, China) and then subjected to reactions with reverse transcriptase (Promega, Madison, WI, USA) to synthesize the single-strand cDNAs. Subsequently, expression of the indicated bZIP genes at mRNA levels in different cell lines were measured by qRT-PCR with distinct pairs of primers as listed in Table S1. The polymerase chain reactions were carried out in the GoTaq^®^ real-time PCR detection systems, loaded on a CFX96 instrument (Bio-rad, Hercules, CA, USA). The results were analyzed by the Bio-Rad CFX Manager 3.0 software (Bio-rad).

### 4.7. The Pulse-Chase Experiments Followed by Western Blotting

After reaching 70% confluence of HepG2 cells that had been allowed for growth in 6-well plates for 24 h in DMEM containing 25 mmol/L glucose and 10% FBS, they were transfected with an expression construct for Nrf1, Nrf2, Nach2, Nach1, or its mutants (each of them was C-terminally tagged by the V5 epitope) in a mixture of Lipofectamine 3000 (Invitrogen Ltd., Carlsbad, CA, USA). After transfection for 24 h, the cells were or were not treated with CHX (at 50 µg/mL) alone or plus MG132 (at 10 µmol/L) for additional 30 min to 4 h before being harvested in a lysis buffer [38]. Total cell lysates were subjected to protein separation by SDS-PAGE gels containing 10% polyacrylamide, followed by Western blotting with antibodies against the V5 epitope (Invitrogen Ltd.) or β-Actin (from Zhong shan Jin qiao Co, Beijing, China). β-Actin served as an internal control to verify amounts of proteins that were loaded in each well occasion. The intensity of Nach1 and its mutant protein bands were quantified and shown graphically.

### 4.8. ARE-Driven Reporter Gene Assays

Equal numbers (1.5 × 10^5^) of HepG2 cells were allowed for 24-h growth in each well of 12-well plates containing DMEM supplemented with 25 mmol/L glucose and 10% FBS. After reaching 70% confluence, the cells were transfected with expression constructs for Nrf1, Nrf2, Nach2, Nach1, or its mutants alone or in different combinations with one another, together with both *GSTA2-6×ARE-Luc* reporter and pRL-TK (as an internal control), in a mixture of Lipofectamine 3000 (Invitrogen Ltd.). Approximately 24 h after transfection, ARE-driven luciferase reporter activity was measured by Magellan7.1 SP1 systems and then calculated as fold changes (mean ± S.D), as described previously [81]. The data presented each represent at least three independent experiments.

### 4.9. Statistical Analysis

Statistical significances of fold changes in the *GSTA2-6×ARE-Luc* reporter activity and also in the gene expression were determined using the Student’s *t*-test or Multiple Analysis of Variations (MANOVA). The data are shown as a fold change (mean ± S.D), each of which represents at least 3 independent experiments that were each performed in triplicates.

## 5. Conclusions

In an attempt to provide a better understanding of the origin of bZIP transcription factors with different subfamily evolutionary features (particularly, the CNC-bZIP subfamily), distinct sizes of four neighbor-joining phylogenetic trees have been established here, based on different conditional grounds. Among them, the first phylogenetic tree (Figure 1c) has been constructed by employing the highly conserved BRLZ domains of total 441 bZIP proteins from 23 representative organisms, including metazoa, protozoa, bacteria, and viruses. This work deciphers the early origin of such a large bZIP superfamily, which is inferred to originate from putative infected viral and/or bacterial cognate genes transferred to the last common ancestor, that had long evolved from prokaryotic to eukaryotic genes and also undergone the natural selection with multiple independent expansions during its evolutionary process. This is indeed supported by the evidence that homologues of both the Jun and BATF subfamilies are identified in viruses, the Jun and C/EBP subfamilies share a big clade of evolutionary tree, and that the C/EBP and CNC-bZIP subfamilies are further diversified inferably from marine bacteria. The further evidence also reveals that expansion and diversification of the bZIP superfamily have occurred in vertebrates from metazoa. The second phylogenetic tree (Figure 2a) has been built with all the full-length Nach/CNC-bZIP proteins, which retain a founding bZIP-adjacent CNC domain so as to enhance their DNA-binding activity of target genes. This work leads to a novel discovery of the Nach subgroup, which is budded at the early evolutionary stage of the CNC-bZIP subfamily transcription factors that have been selected from the marine bacteria to humans. The third phylogenetic tree (Figure 3a) is depicted by all putative TM-containing bZIP and bHLH-ZIP transcription factors. Different TM properties determine distinct topological folding of transmembrane-bound transcription factors within and around the ER, and their further proteolytic processing before being translocated into the nucleus to regulate cognate target genes. Thereby, we here proposed distinct membrane-topobiological models (as illustrated in Figure 3e), based on the data published previously by us and other groups (31–37). Of note, the topobiology of Nrf1, as a representative of the membrane-bound Nach/CNC-bZIP factors, is distinctive from those of XBP1u, ATF6 and SREBP. The fourth phylogenetic tree (Figure 4a) is recapitulated with the intact human bZIP proteins, further revealing that the CNC-bZIP subfamily also shares a common evolutionary progenitor with the Jun group. Importantly, this study has also emphasized that Nrf1α is involved in the complex interaction networks of bZIP proteins in humans, because its functional loss or gain leads to significant alterations in the expression of other bZIP genes. 

Overall, Nach1 to Nach8 are herein identified to comprise a novel subgroup of the CNC-bZIP subfamily, of which the marine bacterial Nach1 and Nach2 share higher homology with human activators of NF-E2 p45, Nrf1 and Nrf2, as well the repressor Bach1. As such, they indeed perform distinct transcriptional abilities to mediate differential expression of ARE-battery genes. Yet, it is required for further determination of a *bona fide* function of Nach1/2 in the marine bacteria and its host animals.

## Supplementary Materials

Supplementary materials can be found at http://www.mdpi.com/1422-0067/19/10/2927/s1. All data needed to evaluate the conclusions in the paper are present in the paper and/or the Supplementary Materials. Additional data related to this paper may be requested from the authors.
