# Supplementary material for: Nach Is a Novel Subgroup at an Early Evolutionary Stage of the CNC-bZIP Subfamily Transcription Factors from the Marine Bacteria to Humans
Source: Int J Mol Sci. 2018 Sep 26;19(10):2927. doi: 10.3390/ijms19102927 (PMC6213907; doi:10.3390/ijms19102927)
Supplement: Supplementary file 1 [file ijms-19-02927-s001.pdf]

International Journal of
Molecular Sciences

MDPI

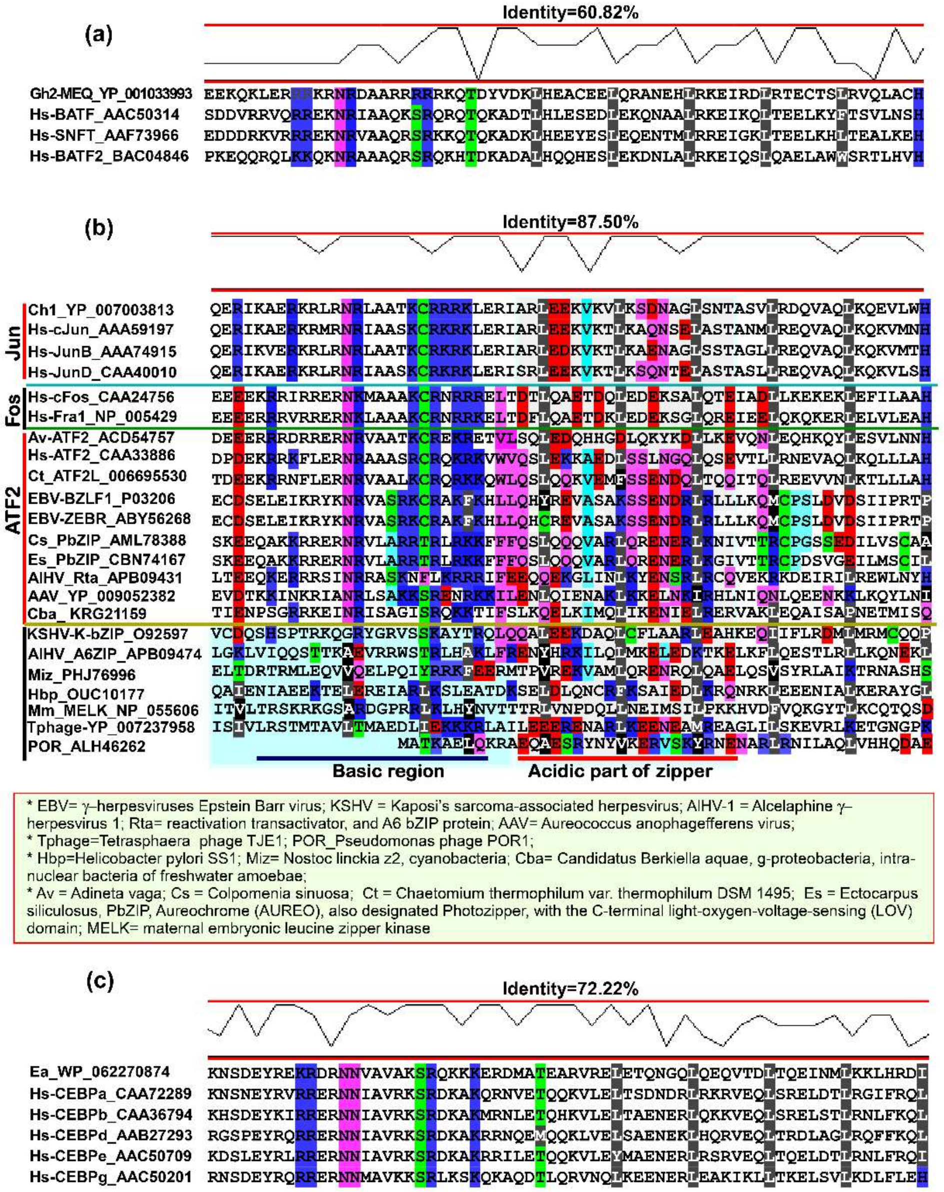

Figure S1. Identity of original homologues of BATF, Jun and C/EBP subfamilies. The multiple sequences alignments of BRLZ domains were analyzed by the DNAMAN8.0 software: (**a**) MEQ from *Gallid herpesvirus 2* is homologous with human BATF family. (**b**) Another viral protein (with a GenBank No. YP_007003813) from *Cyprinid herpesvirus 1* is conserved with human Jun family, including Fos, ATF2 and others. (**c**) A bacterial homologous protein (with a GenBank accession No. WP_062270874) from *Endozoicomonas arenosclerae* is classified into human C/EBP family.

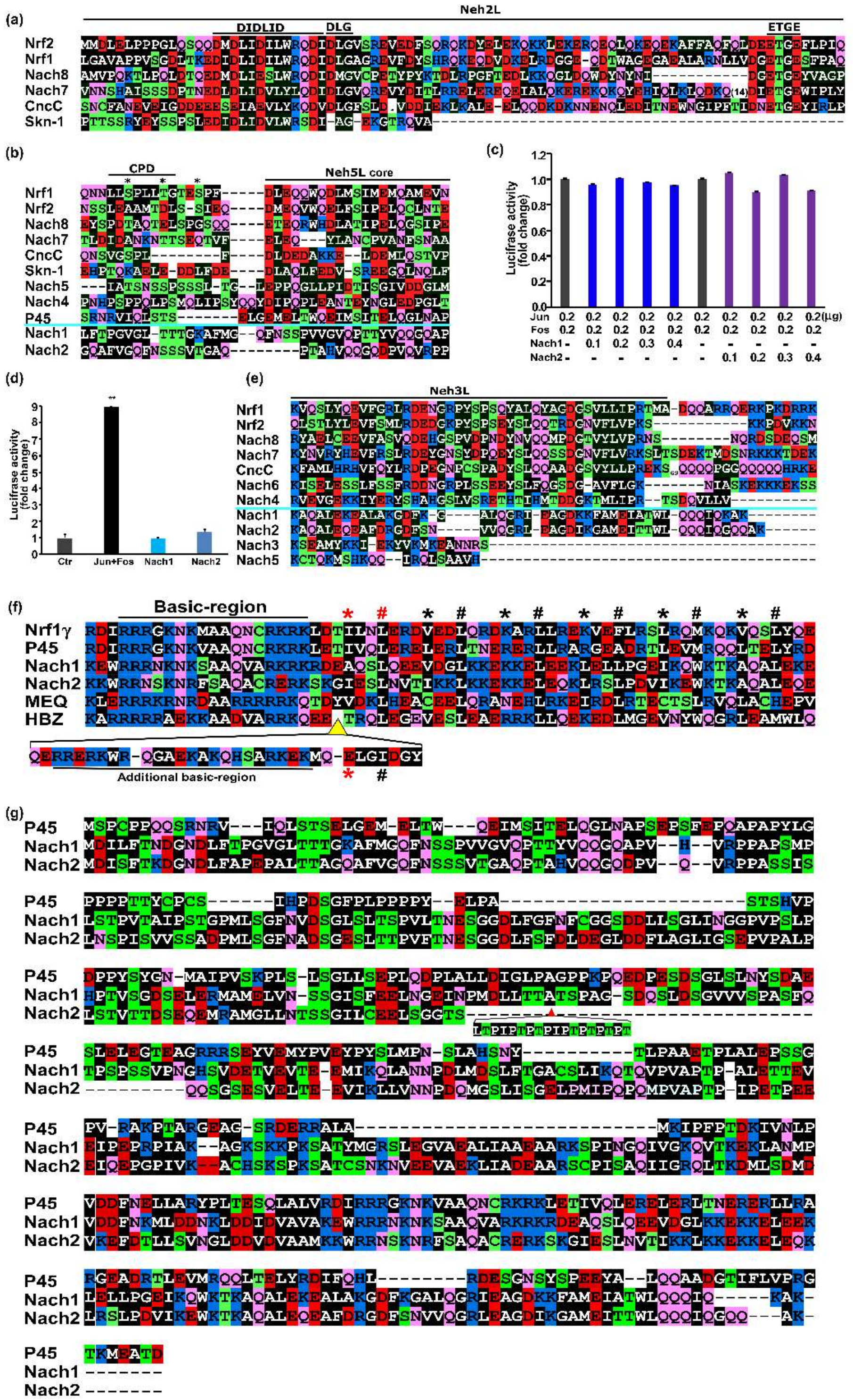

Figure S2. Detailed schematic representation of structural domains of Nach/CNC-bZIP proteins. Bioinformatic analysis by the DNAMAN8.0 software was subject to multiple sequence alignments of different structural domains of: (**a**) the Neh2L domain, (**b**) the Neh5L domain, (**c**) Neh3L domain. (**d**,**e**) Nach1 and Nach2 have no effects on basal expression of AP-1-driven reporter gene and its regulation by Fos and Jun. Related methods and data calculations were referenced to determination of ARE-driven luciferase reporter activity as described in the legend of main Fig. 5. (**f**) Shows specific sequence alignments of the BRLZ domains of HBZ from *Human T-Cell Leukemia Virus Type 1*, MEQ from *Gallid herpesvirus 2*, bacterial Nach1/2 with human Nrf1γ. The symbols * and # represent the "*a*" and "*d*" positions in heptad repeats of LZ region, respectively. (**g**) Shows an additional alignment of the full length Nach1/2 proteins with human NF-E2 P45.

Figures S3–S13. Distinct characteristics of BRLZ domains within different bZIP subfamilies. Those distinct characteristics of BRLZ domain were analyzed by using three different softwares DNAMAN8.0, MEME and Web-logo with default parameters.

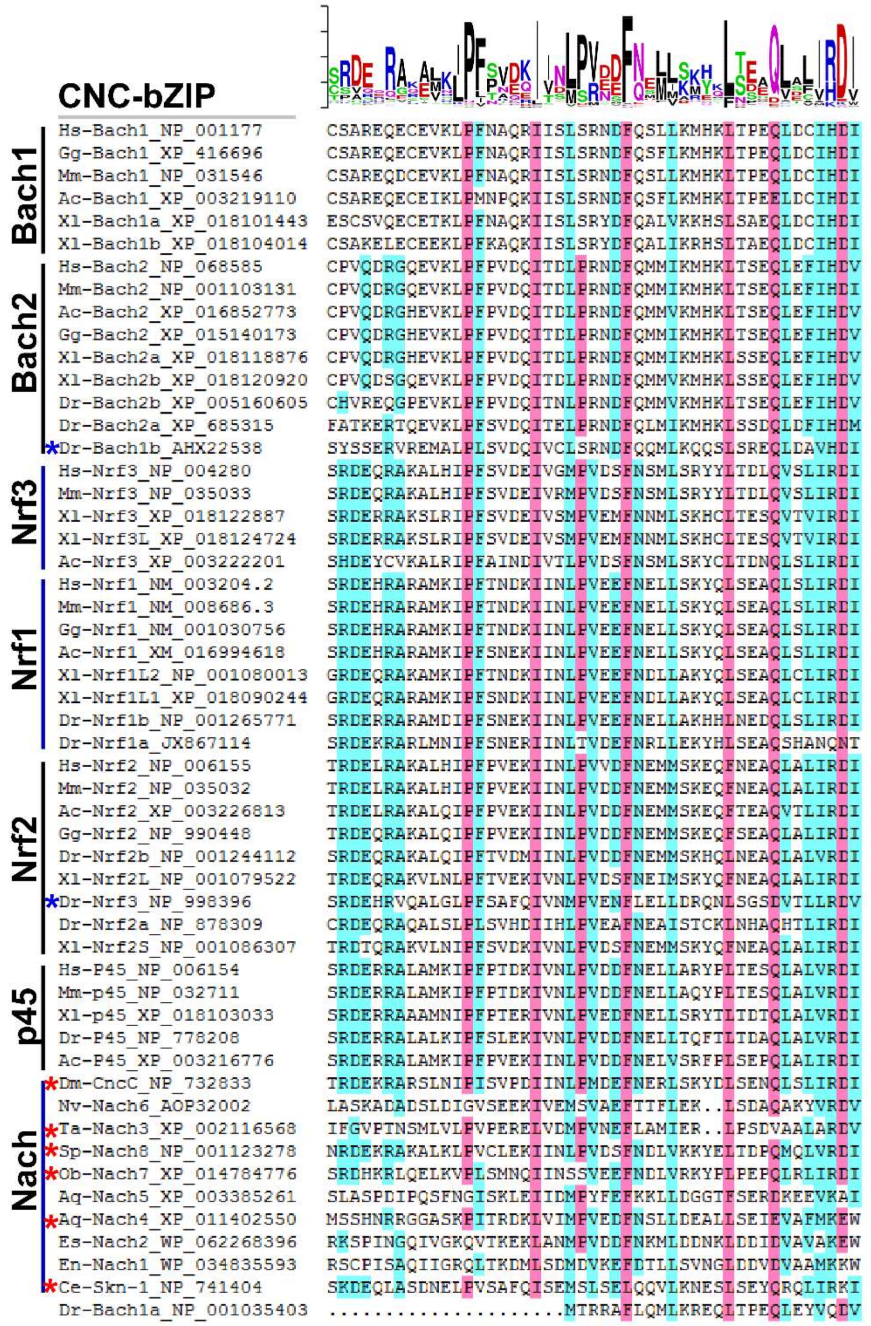

Figure S3. Alignment of the CNC domains from those identified Nach/CNC-bZIP subfamily proteins. The blue and red asterisks represent not gregarious bZIPs in zebrafish and some CNC members with high homology beyond vertebrates, respectively. The light blue and pink backgrounds are 50% to 75% and 75% to 100% homology level.

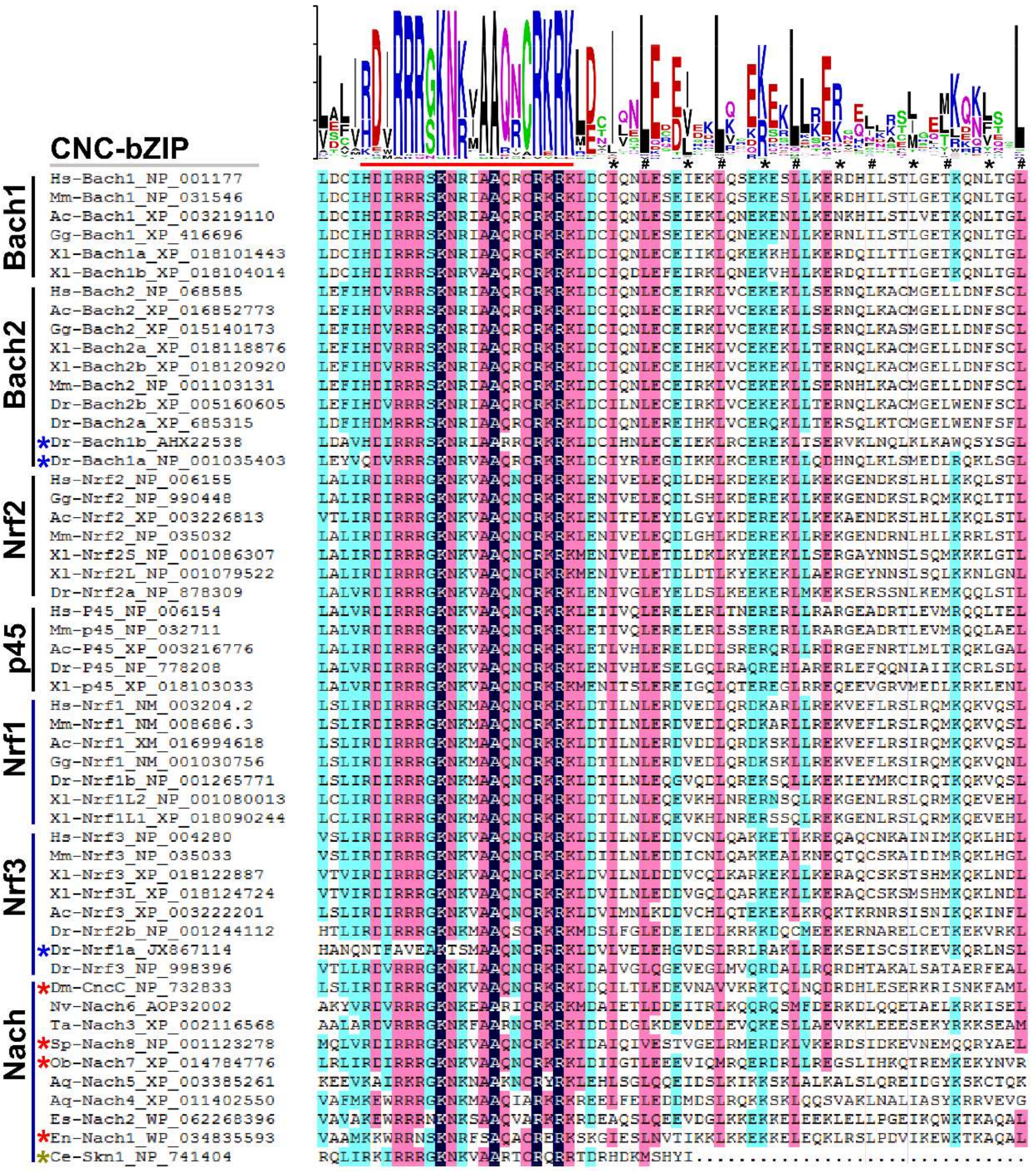

Figure S4. Alignment of the BRLZ domains from those identified Nach/CNC-bZIP subfamily proteins. The blue and red asterisks represent not gregarious bZIPs in zebrafish and some CNC members with high homology beyond vertebrates, respectively. The black symbols * and # represent the "a" and "d" positions in heptad repeats of LZ region, respectively. The light blue, pink and black backgrounds are 50% to 75%, 75% to 100% and 100% homology level.

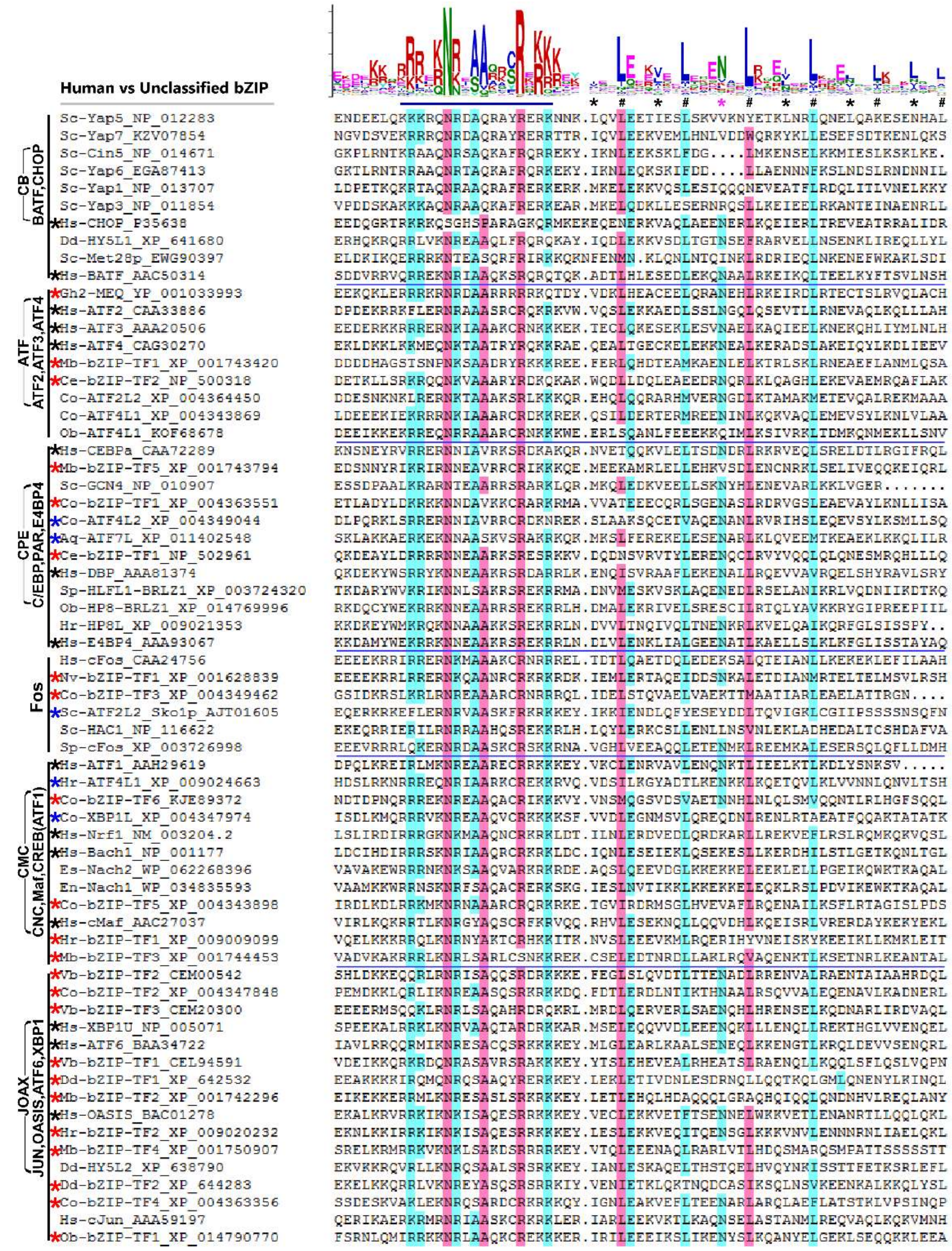

Figure S5. Alignment of the BRLZ domains of unclassified bZIP proteins with human bZIP representatives. The blue, red and black asterisks represent not gregarious bZIPs, unnamed and representative bZIPs, respectively. The black symbols * and # represent the “a” and “d” positions in heptad repeats of LZ region, respectively. The light blue and pink backgrounds are 50% to 75%, 75% to 100% homology level. The Blue lines are corresponding to the classification on the left.

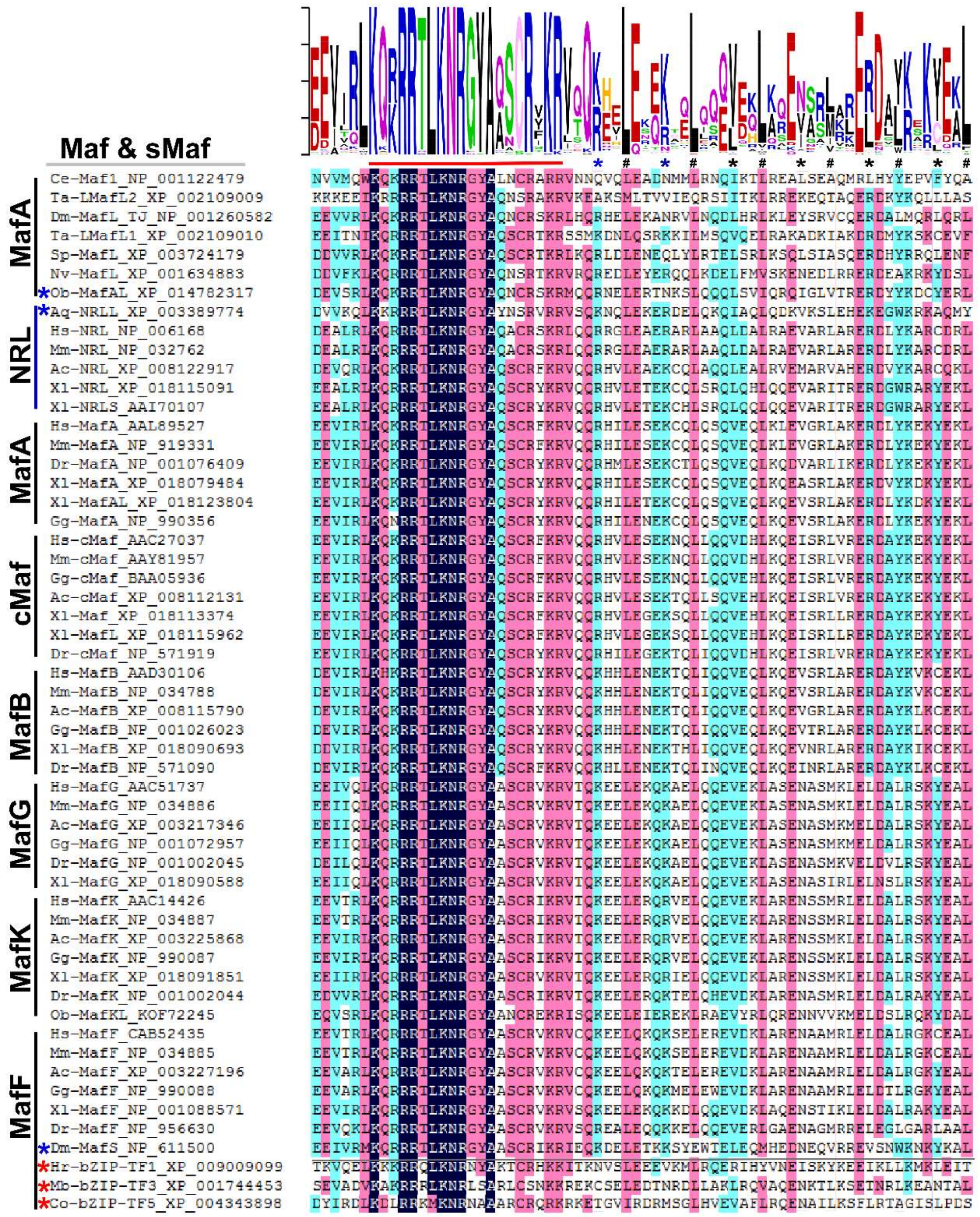

Figure S6. Alignment of the BRLZ domains from within both Maf and sMaf subfamilies. The left blue and red asterisks represent interesting Mafs and unnamed bZIPs, respectively. The black symbols * and # represent the "a" and "d" positions in heptad repeats of LZ region, respectively, where the blue symbols * are the first and second repeat "a" position with basic amino acids. The light blue, pink and black backgrounds are 50% to 75%, 75% to 100% and 100% homology level.

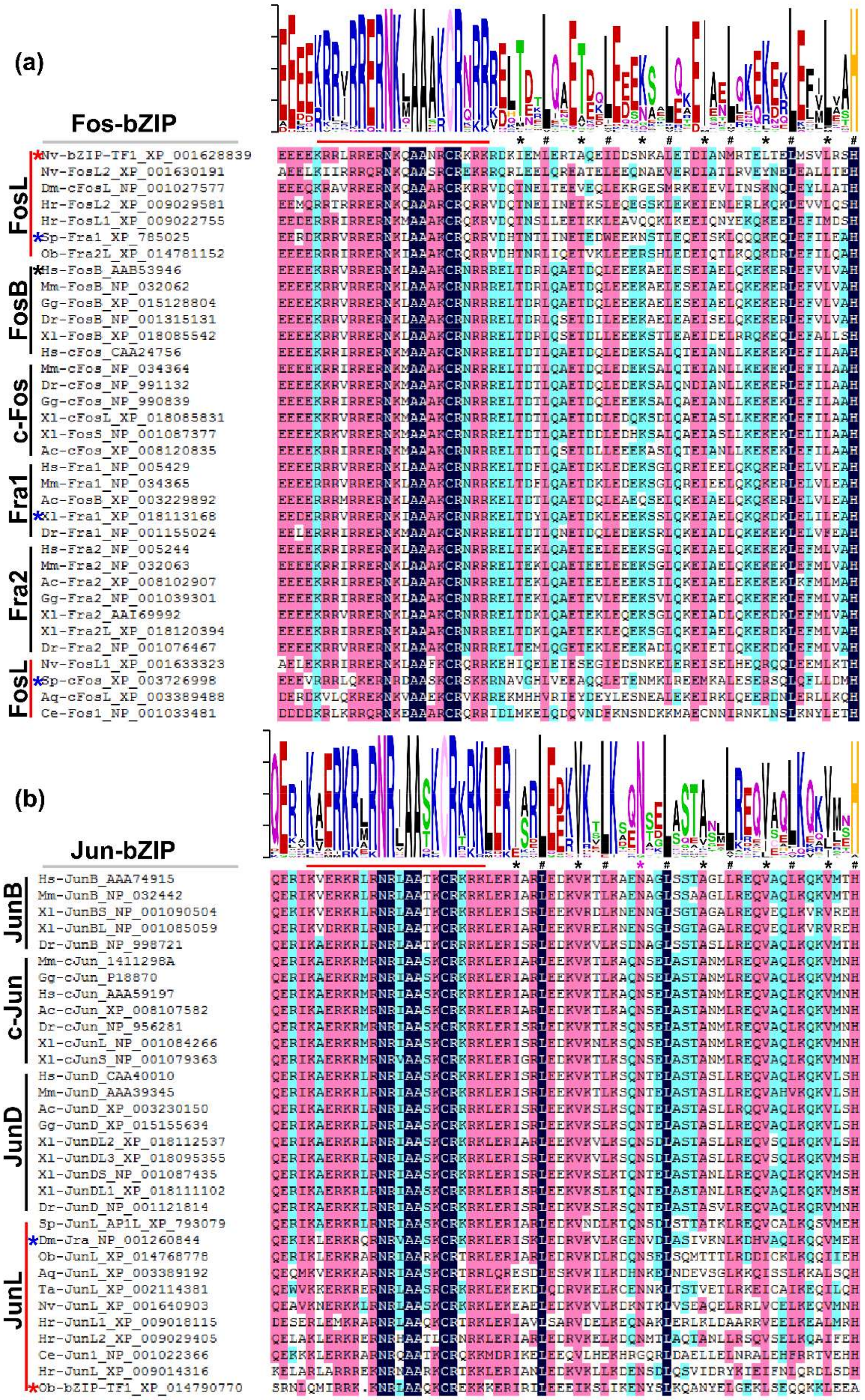

Figure S7. Alignment of the BRLZ domains from within both Fos (a) and Jun (b) subfamilies. The left blue and red and black sterisks represent interesting bZIPs, unnamed and representative bZIPs, respectively. The black symbols * and # represent the "a" and "d" positions in heptad repeats of LZ region, respectively. The light blue, pink and black backgrounds are 50% to 75%, 75% to 100% and 100% homology level.

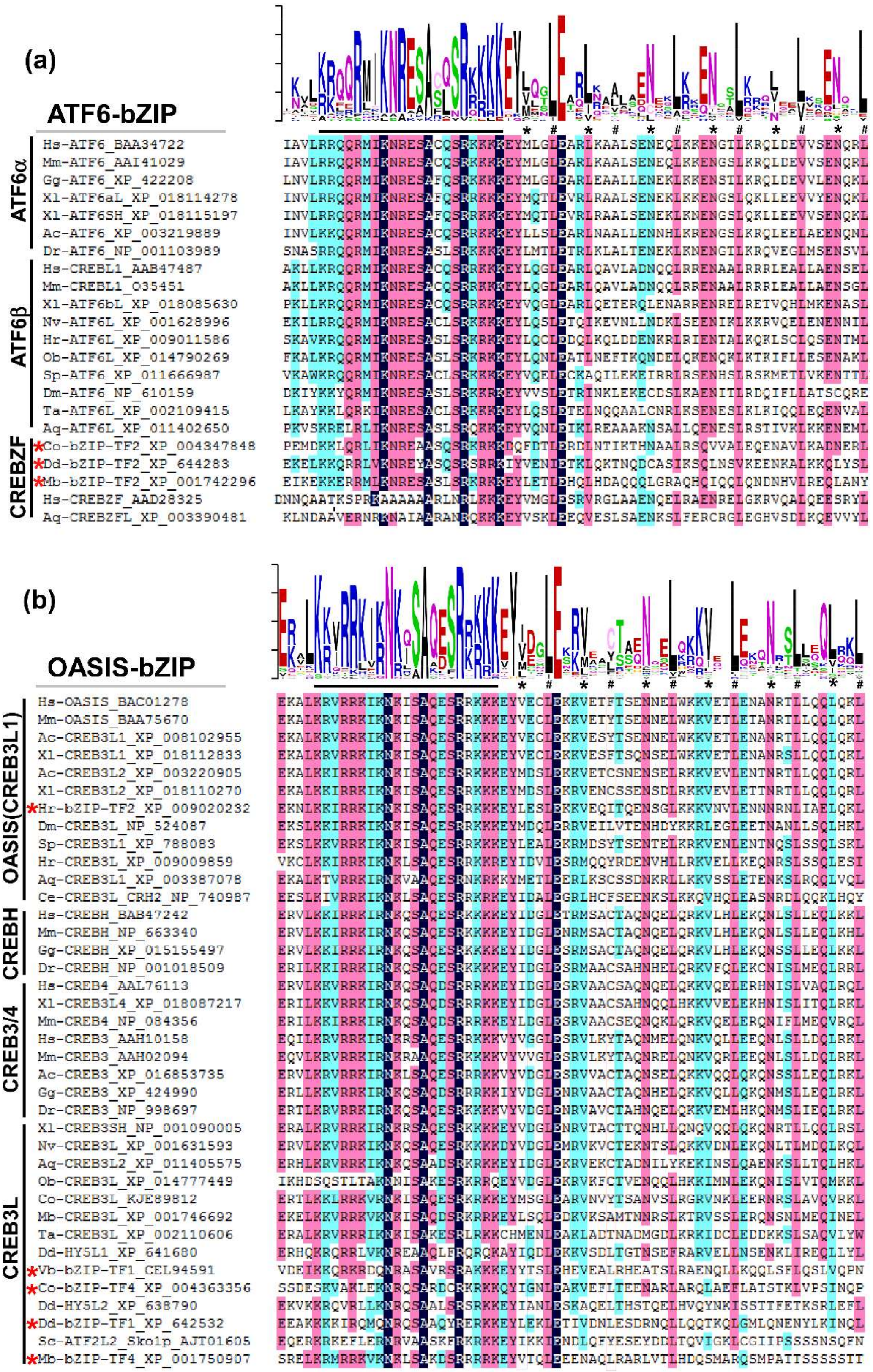

Figure S8. Alignment of the BRLZ domains from within both ATF6 (a) and OASIS (b) subfamilies. The left red asterisks represent unnamed bZIPs. The black symbols * and # represent the "a" and "d" positions in heptad repeats of LZ region, respectively. The light blue, pink and black backgrounds are 50% to 75%, 75% to 100% and 100% homology level.

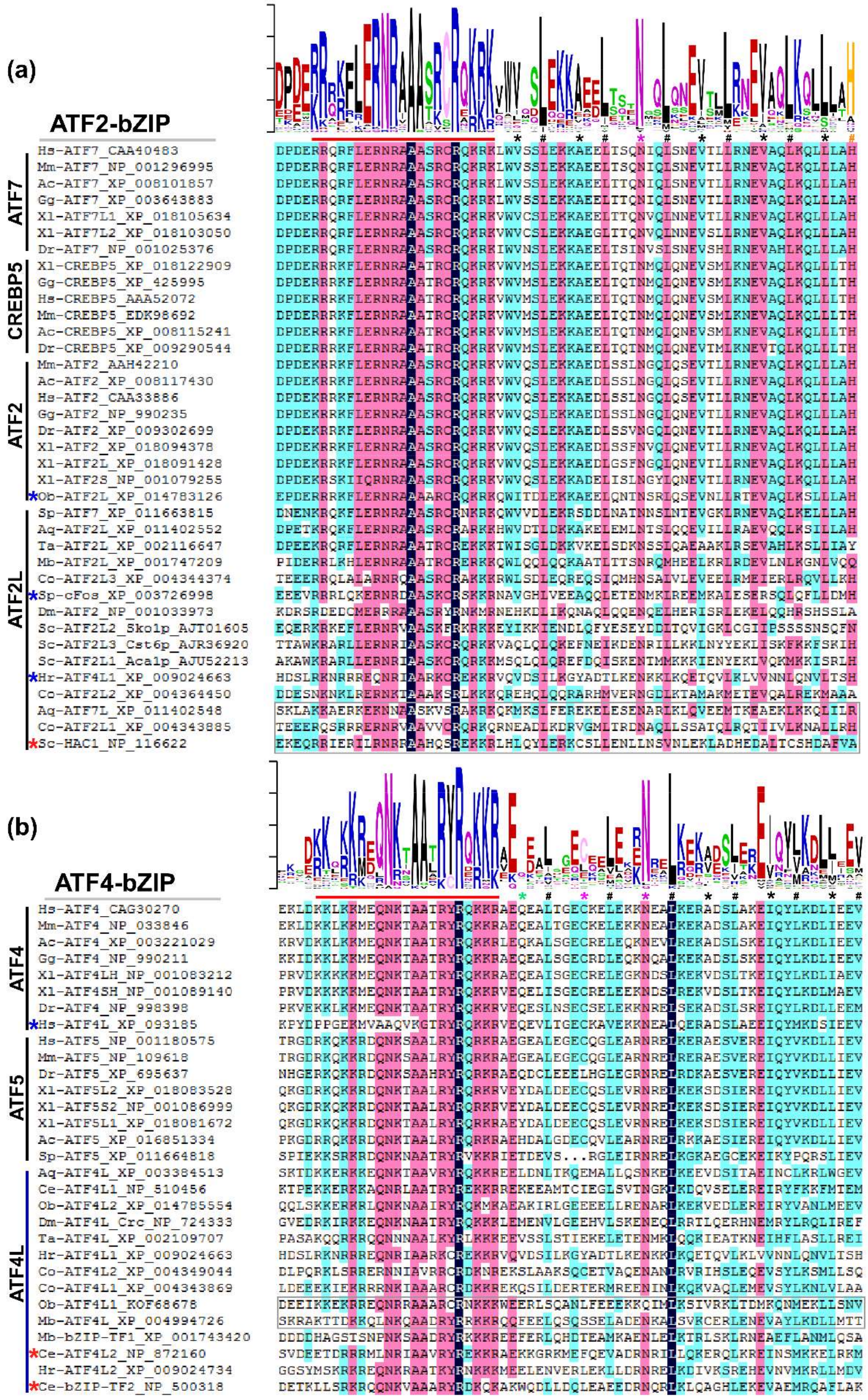

Figure S9. Alignment of the BRLZ domains from within both ATF2 (a) and ATF4 (b) subfamilies. The left blue and red asterisks represent interesting bZIPs and unnamed bZIPs, respectively. The different colors above symbols * and # represent the "a" and "d" positions in heptad repeats of LZ region, respectively. The light blue, pink and black backgrounds are 50% to 75%, 75% to 100% and 100% homology level.

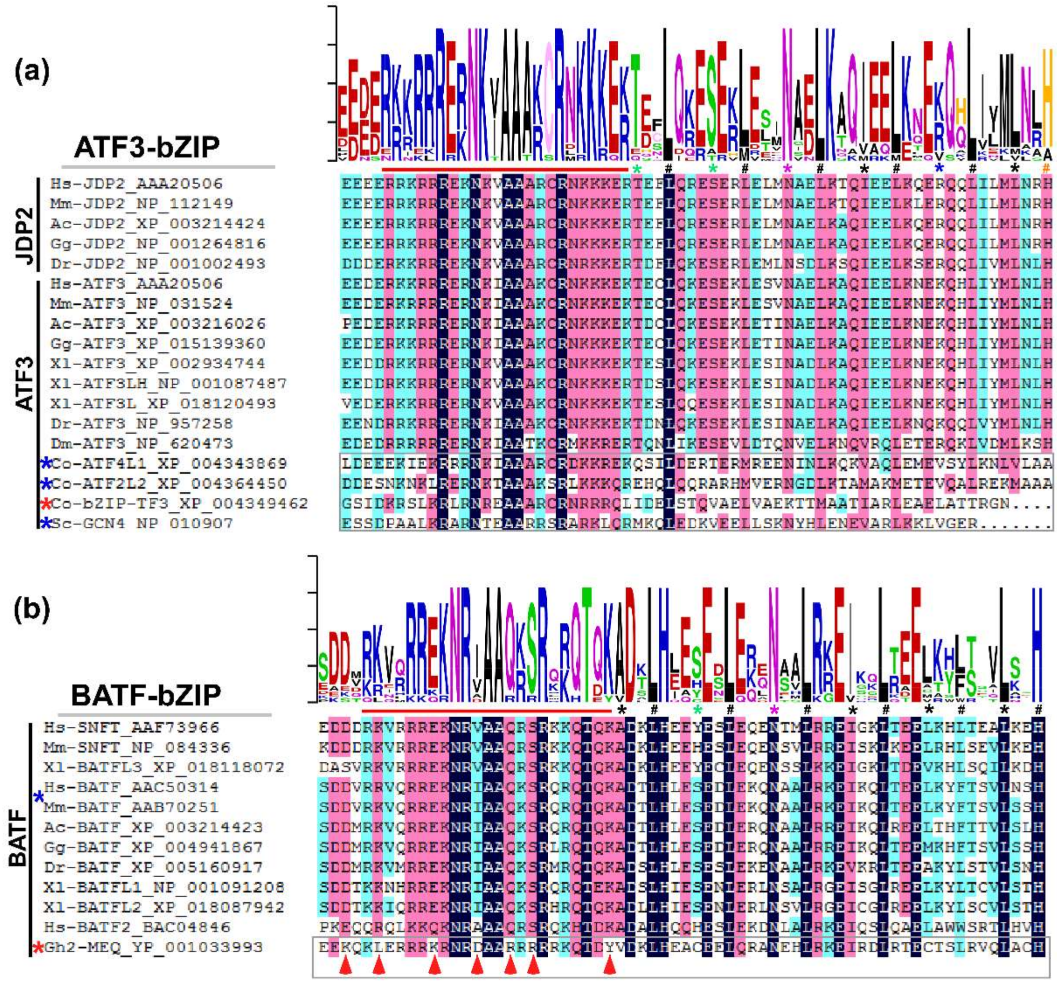

Figure S10. Alignment of the BRLZ domains from within both ATF3 (a) and BATF (b) subfamilies. The left blue and red asterisks represent interesting bZIPs and unnamed bZIPs, respectively. The different colors above symbols * and # represent the "a" and "d" positions in heptad repeats of LZ region, respectively. The red triangles represent the amino acids in Gh2-MEQ is different from others. The light blue, pink and black backgrounds are 50% to 75%, 75% to 100% and 100% homology level.

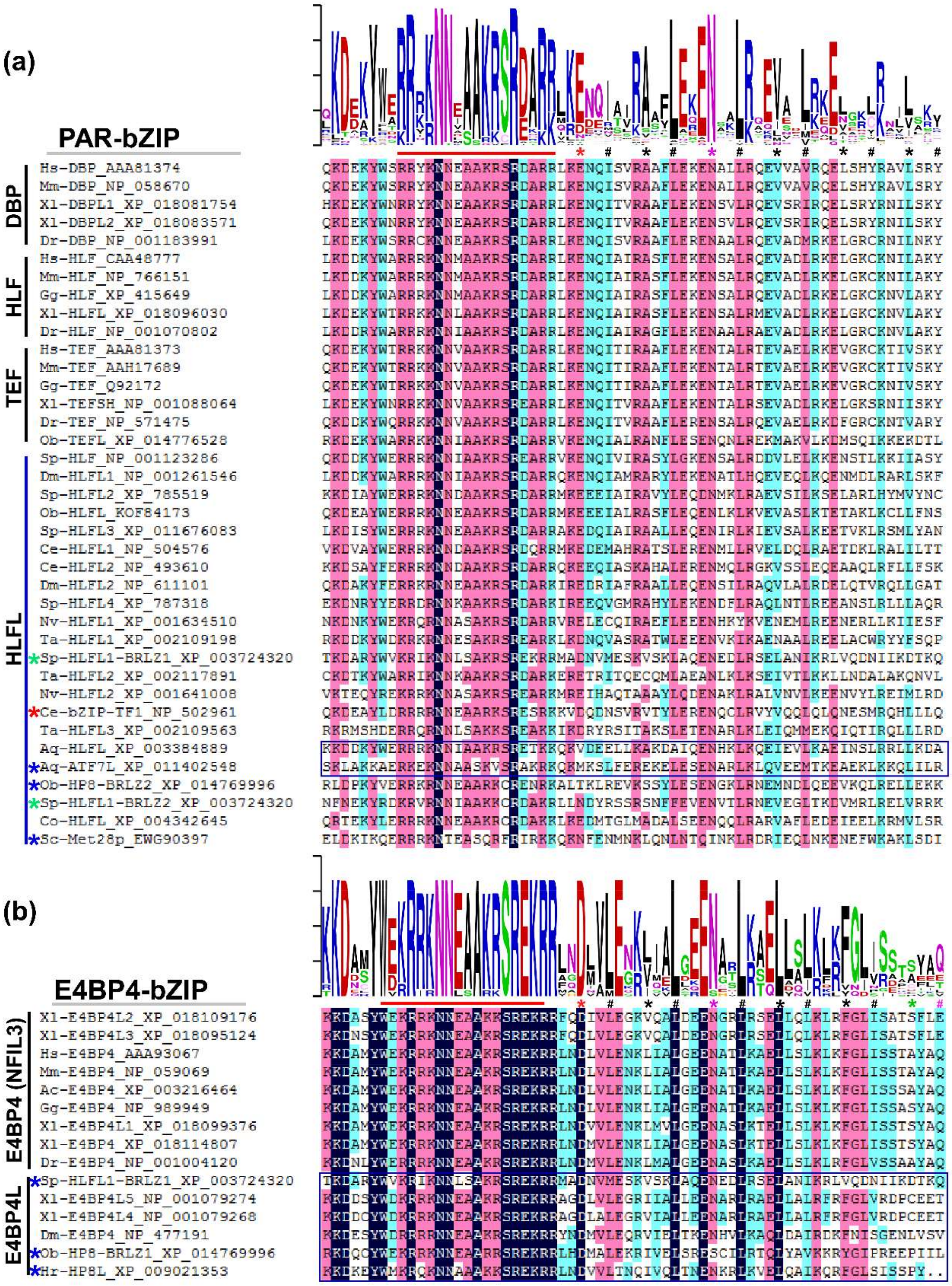

Figure S11. Alignment of the BRLZ domains from within both PAR (a) and E4BP4 (b) subfamilies. The left blue, green and red asterisks represent not gregarious, contained two BRLZ domains and unnamed bZIPs, respectively. The different colors above symbols * and # represent the "a" and "d" positions in heptad repeats of LZ region, respectively. The blue boxes represent highly similar bZIPs, the red lines represent the nuclear localization signal (NLS) in the basic region. The light blue, pink and black backgrounds are 50% to 75%, 75% to 100% and 100% homology level.

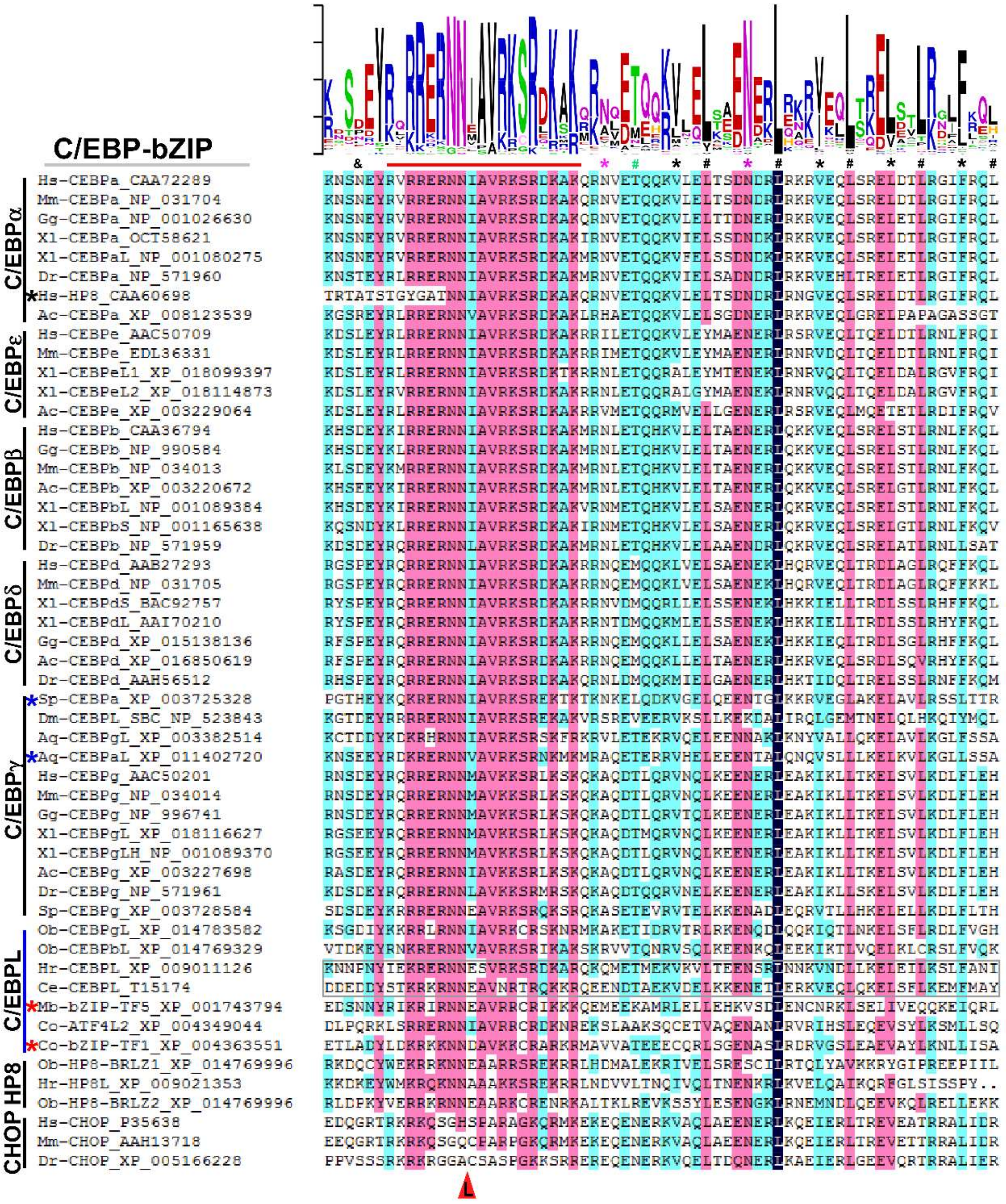

Figure S12. Alignment of the BRLZ domains from within the C/EBP subfamilies. The left blue and red and black asterisks represent interesting, unnamed and representative bZIPs, respectively. The different colors above symbols * and # represent the "a" and "d" positions in heptad repeats of LZ region, respectively. The grey boxes represent highly similar bZIPs, the red triangle represents a cumbrous leucine in Dr-CHOP. The light blue, pink and black backgrounds are 50% to 75%, 75% to 100% and 100% homology level.

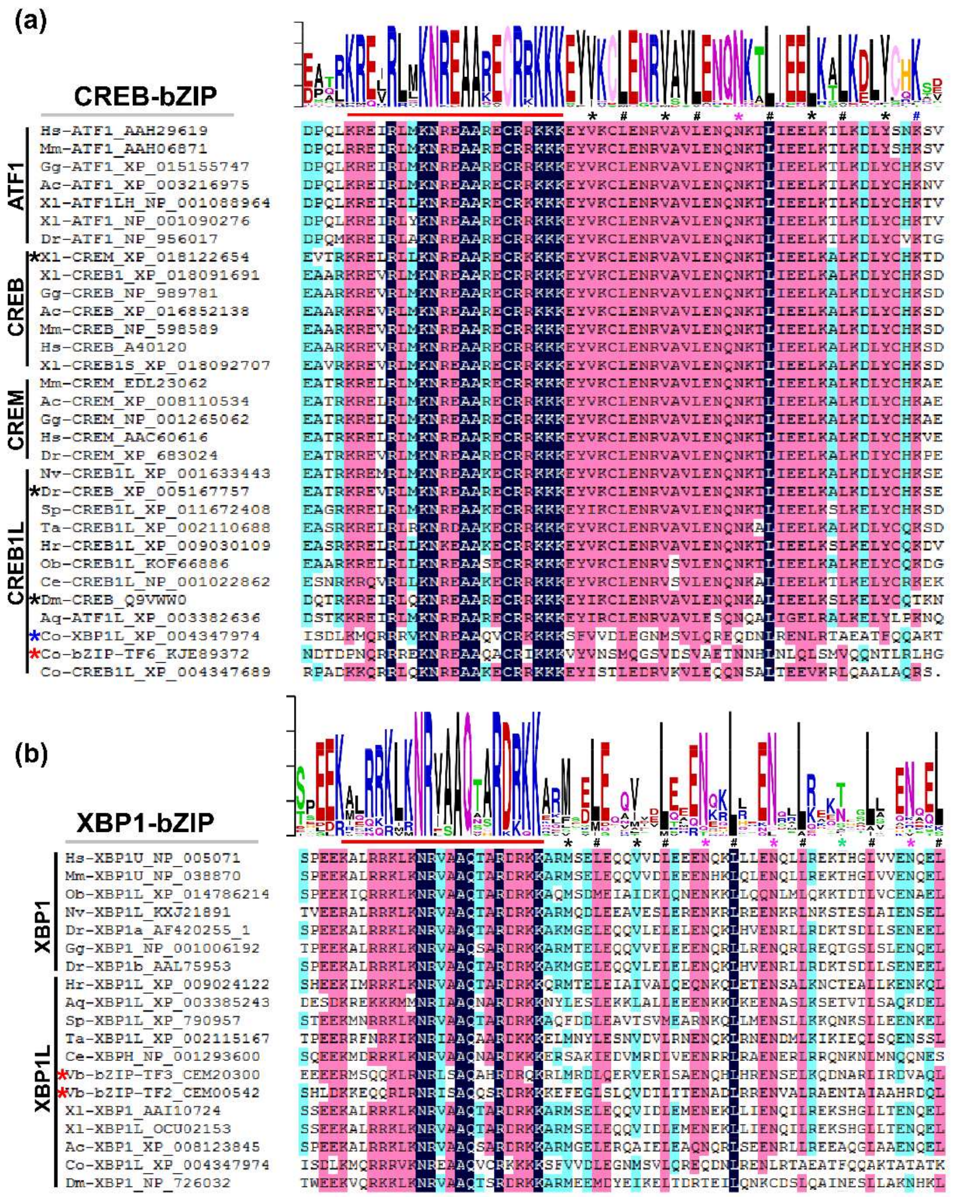

Figure S13. Alignment of the BRLZ domains from within both CREB (a) and XBP1 (b) subfamilies. The left blue, red and black asterisks represent interesting, unnamed and representative bZIPs, respectively. The different colors above symbols * and # represent the "a" and "d" positions in heptad repeats of LZ region, respectively. The light blue, pink and black backgrounds are 50% to 75%, 75% to 100% and 100% homology level.

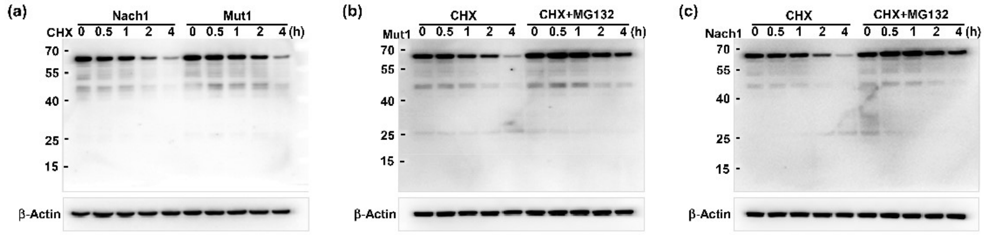

Figure S14. The whole images of figure 5I. (**a** to **c**) Western blotting of Nach1 and its Mut1 that had been resolved by the whole PAGE gels containing 10% polyacrylamide, of which the cropped images were also shown in the main i1, i2 and i3 in Figure 5i, respectively.

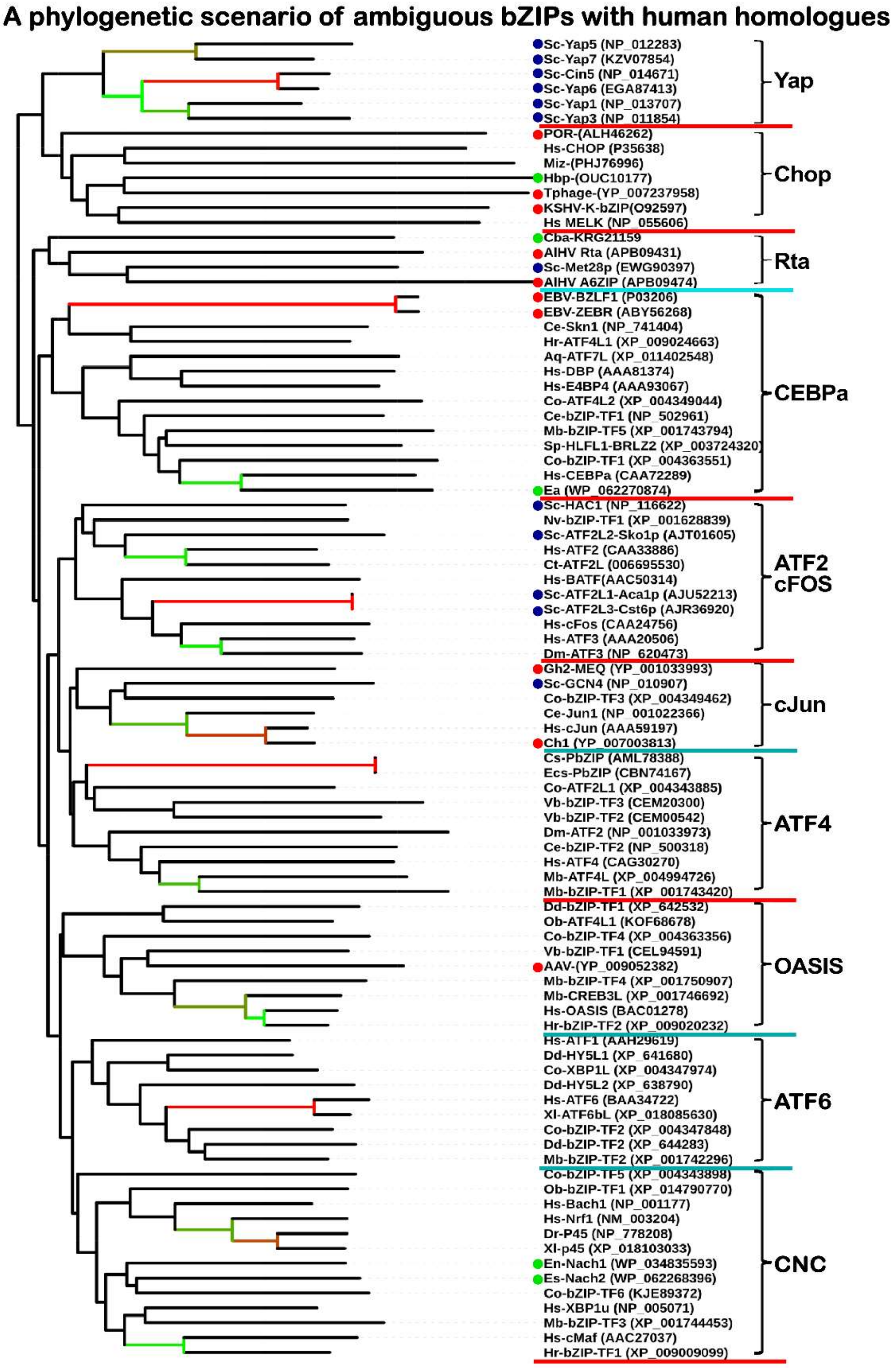

Figure S15. The phylogenetic analysis of unclassified bZIP proteins with human bZIP representatives. The dots with different color are interesting bZIPs, the lines with different color are corresponding to the classification on the right.

Table S1. The primers for qRT-PCR analysis and expression plasmids.

Name	Forward (5'-3')	Reverse (5'-3')
**1, The following primers used for expression plasmids**		
Nach1	CGGGATCCATGGATATTTTGTTTA CAAATGATGGC	CCGCTCGAGCTTTTGCTTTCTGAATT TGCTGCTGTAGCC
Nach2	CGGGATCCATGGATATTTCGTTTACA AAGGATGGCAAT	CCGCTCGAGCCTTTGCTTGTTGCCC CTGAATCTGCT
Mut1	CCATGCTGTCAGGATTCAATGTAACT AGTCCTGTTCTTACTAATGAAAG	CTTTCATTAGTAAGAACAGGACTAGT TACATTGAATCCTGACAGCATGG
Mut2	GATTCAATGTAGACGCCGGACTAGC ACTGACTAGTCCTG	CAGGACTAGTCAGTGCTAGTCCGGC GTCTACATTGAATC
**2, The following primers used for qRT-PCR analysis**		
Nrf1	GGAGGAGTTCAATGAACTGCTGTC	CTCTGGACCTTCTGCTTCATCTGT
ATF1	AGGACTCATCCGACAGCATAG	TTCTGCCCCGTGTATCTTCAG
ATF2	GCACAGCCCACATCAGCTATT	GGTGCCTGGGTGATTACAGT
ATF3	GCTAACCTGACGCCCTTTGT	TTCAGTTCAGCATTCACACTTTCC
ATF4	CCCTTCACCTTCTTACAACCTC	TGCCCAGCTCTAAACTAAAGGA
CHOP	GGAAACAGAGTGGTCATTCCC	CTGCTTGAGCCGTTCATTCTC
DBP	AGCTGATCTTGCCCTATCAAGCA	TTGGCTGCCTCGTTGTTCTTGTA
JUND	TCATCATCCAGTCCAACGGG	TTCTGCTTGTGTAAATCCTCCAG
MAFF	GTGTGCCAGAAGGAGGAGCT	TTGACGATGGTGATGACGCT
NRL	GGCTCCACACCTTACAGCTC	GGCCCATCAACAGGGACTG
β-actin	CATGTACGTTGCTATCCAGGC	CTCCTTAATGTCACGCACGAT
